# Cyanobacterial redox carriers support photosynthesis in a purple phototrophic bacterium

**DOI:** 10.1042/BCJ20253114

**Published:** 2025-08-13

**Authors:** Adam G.M. Bowie, Andrew Hitchcock, Matthew S. Proctor, Elizabeth C. Martin, David J.K. Swainsbury, C. Neil Hunter

**Affiliations:** 1 Plants, Photosynthesis and Soil, School of Biosciences, University of Sheffield, Sheffield, UK; 2 Molecular Microbiology: Biochemistry to Disease, School of Biosciences, University of Sheffield, Sheffield, UK; 3 School of Biological Sciences, University of East Anglia, Norwich Research Park, Norwich, UK

**Keywords:** cytochromes, photosynthesis, plastocyanin, RC-LH1, *Rhodobacter sphaeroides*, *Synechocystis*sp. PCC 6803

## Abstract

In oxygenic and anoxygenic photosynthesis, excitation energy migrates from a surrounding antenna to specialised chlorophyll (Chl) or bacteriochlorophyll (BChl) pigments housed within a reaction centre (RC) complex. Here, a charge-separated state is formed within a few picoseconds, and an electron moves along a series of cofactors until it arrives at a quinone or iron–sulphur centre acceptor. Further photochemical cycles rely on rapid re-reduction in the photo-oxidised RC, usually by small, soluble metalloproteins which vary considerably between different phototrophic clades. In the purple phototrophic bacterium *Rhodobacter* (*Rba*.) *sphaeroides*, the electron carrier cytochrome *c*_2_ (cyt *c*_2_) shuttles between the periplasmic faces of the cytochrome *bc*_1_ complex and the reaction centre-light harvesting 1 (RC-LH1) core complex, the location of the BChl special pair (P_865_). By contrast, in the model cyanobacterium *Synechocystis* sp. PCC 6803, electrons are transferred from cytochrome *b*_6_ *f* to photosystem I (PSI) via two isofunctional redox carrier proteins, cytochrome *c*_6_ (cyt *c*_6_) or plastocyanin (Pc). In the present study, we demonstrate that both cyt *c*_6_ and Pc can substitute for cyt *c*_2_ *in silico*, *in vitro* and *in vivo*, even though their electrostatic properties may be counter-productive for binding the RC-LH1 complex. Interestingly, whilst P_865_^+^ reduction was highest with cyt *c*_2_ and the full physiological RC-LH1 complex, both *Synechocystis* proteins were more compatible with the RC-only complex lacking the surrounding LH1 antenna. Taken together, this suggests the subunits that constitute the LH1 ring improve both the donor side activity and selectivity of the *Rba. sphaeroides* RC complex.

## Introduction

Oxygenic and anoxygenic photosynthesis are powered by spectrally distinct reaction centres (RCs) that share a common evolutionary origin [1–4]. This relatedness is evident from the structural homology between the five core transmembrane helices of all extant RCs, but after billions of years of divergent evolution, modern RC complexes share little meaningful sequence identity [2]. Some RCs use bacteriochlorophylls (BChls) as their primary charge-separating pigments, whilst others harness chlorophylls (Chls), but their electron acceptors are the basis for delineating two RC classes. Following photochemical charge separation, Type I (Fe–S type) RCs pass electrons to iron–sulfur clusters via either of the two (pseudo)symmetric branches of cofactors, whereas type II (Q-type) RCs reduce quinone acceptors via the active branch (A-branch) of cofactors [1]. There are also differences in the way electrons are supplied to the photo-oxidised primary pigments, resetting RCs for further rounds of photochemistry. The electron donors to both type I and type II RCs in the photo-oxidised state are generally small, mobile electron carriers such as *c*-type cytochromes or plastocyanin (Pc), with the notable exception of photosystem II (PSII) in cyanobacteria, algae and plants, which has evolved the capacity to extract electrons from water, generating oxygen as a ‘waste’ by-product [5,6]. The green or purple phototrophic bacterial clades make either type I or type II RCs, respectively, and have relatively simple, cyclic photosynthetic electron transfer chains, whereas cyanobacteria, algae and plants evolved the ability to assemble both RC types, specifically photosystem I (PSI) and the water-oxidising PSII complex [7]. Consequently, oxygenic photosynthesis employs a linear electron transfer pathway that couples PSII, cytochrome *b*_6_ *f* (cyt *b*_6_ *f*) and PSI complexes so that electrons from water are eventually used to reduce CO_2_ to simple carbohydrates [8].

Photosynthetic organisms occupy specific spectral niches related to the light-absorbing pigments they produce. Purple phototrophic bacteria synthesising BChl *a* harvest near-infrared light in the 750–950 nm spectral region, whereas oxygenic phototrophs equipped with PSI and PSII use Chls to harvest visible light below 750 nm. This kind of specialisation has led to proposals to combine the attributes of both Chl- and BChl-containing RC complexes in a host organism, generating a hybrid, light-driven electron transfer system with an expanded spectral range able to absorb nearly all photosynthetically active solar radiation [9,10]. The native arrangement of the PSII and PSI complexes, both of which bind Chl *a*, was suggested to be inherently inefficient because both photosystems compete for the same photons [9]. Their vision of re-engineered photosynthesis replaced Chl *a*-containing PSI with a BChl *b*-based RC complex termed ‘RC1′, creating a more efficient photochemical system with complementary light absorption properties and minimal spectral overlap [9]. A subsequent proposal also replaced PSI with a BChl *b*-containing type II RC that drives an electron transfer loop involving the cyt *b*_6_ *f* complex, resembling cyclic electron transport in phototrophic bacteria. This new arrangement would generate a protonmotive force that drives the ATP synthase, whilst NADP^+^ could be reduced by the NAD(P)H dehydrogenase complex operating in reverse [10].

**Figure 1:**
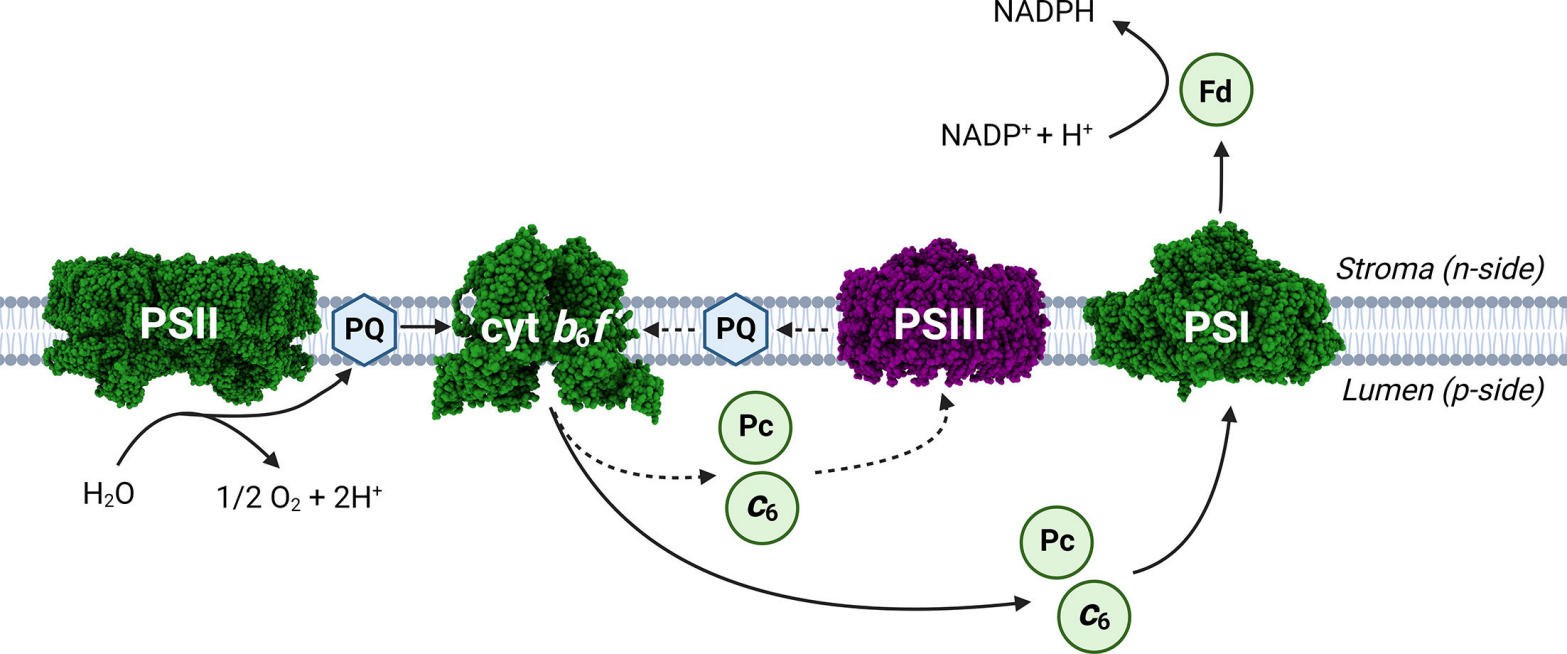
Envisaged three-photosystem electron transport chain in a transgenic cyanobacterium. Electron transport pathways in a ‘photo-redesigned’ thylakoid membrane, as proposed by Hitchcock et al. [11], with native electron transfer proteins depicted in green and a near-infrared absorbing third photosystem (‘PSIII’) based on the reaction centre-light harvesting 1 (RC-LH1) complex of purple bacteria (coloured purple). This heavily redesigned complex would oxidise cytochrome *c*_6_ (cyt *c*_6_)/plastocyanin (Pc) and pass photoexcited electrons to plastoquinone (PQ), forming an additional cyclic electron transfer loop with cytochrome *b*_6_ *f* (cyt *b*_6_ *f* ) (dashed lines) that contributes to thylakoid lumen acidification and ATP production. Space-filling models are included for photosystem I (PSI) [13], photosystem II (PSII) [14], cyt *b*_6_ *f* [15] and RC-LH1 [16] structures; host cyclic electron transfer is omitted for clarity.

A more recent proposal [11,12], depicted in Figure 1, retains the native PSII and PSI photosystems whilst introducing a new BChl *a*-based type II RC complex termed ‘PSIII’, forming a three-photosystem hybrid array that can utilise the visible, red, far-red and near-infrared regions of the solar spectrum. In this arrangement, intended to operate in a minimal cyanobacterial chassis [17], an engineered BChl-PSIII and the native Chl-PSI complex would compete for the same electron donors, cyt *c*_6_ and Pc, with the relative electron flux through the two RCs being determined by whether the chromatic environment preferentially excites either Chl or BChl [11]. A minimally effective PSIII complex could be optimised by using adaptive laboratory evolution (ALE), which has already been used to introduce new traits in photosynthetic organisms such as high-light tolerance [18,19].

The list of modifications and genetic manipulations required to assemble a purple bacterial RC complex in a cyanobacterial chassis is formidable, particularly the heterologous expression of reaction centre-light harvesting 1 (RC-LH1) structural genes alongside their assembly factors and the wholesale rewiring of pigment biosynthesis pathways to make both Chl and BChl simultaneously [11]. Using the well-characterised RC complex from *Rba. sphaeroides* as a starting point for PSIII, extensive protein engineering would also be required to make the complex compatible with the native cyanobacterial photosynthetic machinery, reducing plastoquinone instead of ubiquinone (UQ) and oxidising cyt *c*_6_ and Pc rather than cyt *c*_2_. To investigate the feasibility of a PSIII complex, we have determined the level of pre-existing compatibility at the donor side of the *Rhodobacter* (*Rba.) sphaeroides* RC complex for binding the native cyt *c*_2_ relative to cyt *c*_6_ and Pc counterparts from *Synechocystis* sp. PCC 6803.

PSI and RC-LH1 accept electrons from soluble redox carrier proteins at binding sites that can be divided into two regions with different properties. In both RC types, a short-range interaction domain composed primarily of hydrophobic residues acts as the site of electron transfer, located on a pair of antiparallel helices that lie directly above the primary charge-separating pair of (B)Chls [3,20,21]. This region interacts with the face of the incoming electron donor through a series of van der Waals (vdW) contacts, hydrogen bonds and cation-π interactions, which are centred around a functionally imperative aromatic electron tunnelling contact, Tyr-L162 in RC-LH1 [20,22,23], and a pair of π-stacked tryptophan residues in PSI [24–26]. By contrast, a long-range interaction domain is formed by more distal charged residues making a series of complementary electrostatic interactions that steer the redox carrier protein towards its target via a transient encounter complex [20,27,28]. The balance between these two domains is different in the two RC complexes. The PSI interface is simpler and more symmetrical, being dominated by hydrophobic interactions and two arginine–aspartate charge pairs, whilst the RC-LH1 binding site has a much larger electrostatic component, featuring a ring of amino acids with negatively charged side chains [20,28,29].

The compartmentalisation of hydrophobic and charged residues is also apparent in the structures of the redox carrier proteins. In the class I *c*-type cytochromes cyt *c*_2_ and cyt *c*_6_, uncharged residues border the leading edge of the heme *c* prosthetic group, whilst in the blue copper protein Pc, a non-polar patch of residues known as the ‘northern face’ surrounds His-86, a key amino acid known to be part of the electron transfer pathway to PSI [30–32]. An outer ring of positively charged residues in cyt *c*_2_ makes electrostatic interactions with RC-LH1, whilst cyt *c*_6_ and Pc have acidic patches for interactions with PSI [25,32–34], although the eukaryotic homologues of these two proteins have much greater negative charge density due to electrostatic interactions with PsaF that are absent from cyanobacteria [25,30,35–37]. These differences in donor–acceptor binding interfaces between PSI, RC-LH1 and their respective electron donors should present a significant obstacle to cross-species compatibility, even though the RCs and their respective cytochrome redox carriers share significant structural homology. Here, we investigate the capacity of cyt *c*_6_ and Pc to replace cyt *c*_2_ as the electron donor to RC-LH1 and RC complexes *in silico*, *in vitro* and *in vivo*. We show that cyt *c*_6_ is an adequate replacement for the native cyt *c*_2_ donor *in vivo* despite its intrinsically low RC reduction rate *in vitro*, whilst Pc is a uniformly poor electron donor to RCs in both situations. However, the *in vivo* performance of Pc can be greatly improved by ALE, which has the effect of raising heterologous Pc biosynthesis at least seven-fold. We also identify possible roles for the additional subunits that surround the RC core complex, including the LH1 αβ polypeptides, PufX, protein-Y and protein-Z. Although it is not known which subunits are responsible, the presence of these LH1-associated proteins collectively improves both the activity of the RC complex and its electron donor selectivity.

## Results

### Cyt *c*_6_ and Pc productively bind RC-LH1 in AlphaFold3 predicted complexes

Once bound together in a complex, the rate of electron transfer between two redox-active proteins is determined by a range of highly sensitive parameters, including the difference in midpoint redox potentials that determines the driving force (Δ*G*), the reorganisation energy (λ) and the edge-to-edge distance between two redox active cofactors [38]. Whilst RC-donor systems can tolerate significant changes in Δ*G* values [39], nearly all productive biological electron transfers occur over a narrow distance range of 4–14 Å, making the binding location and orientation of an incoming redox carrier protein functionally imperative [40].

**Figure 2:**
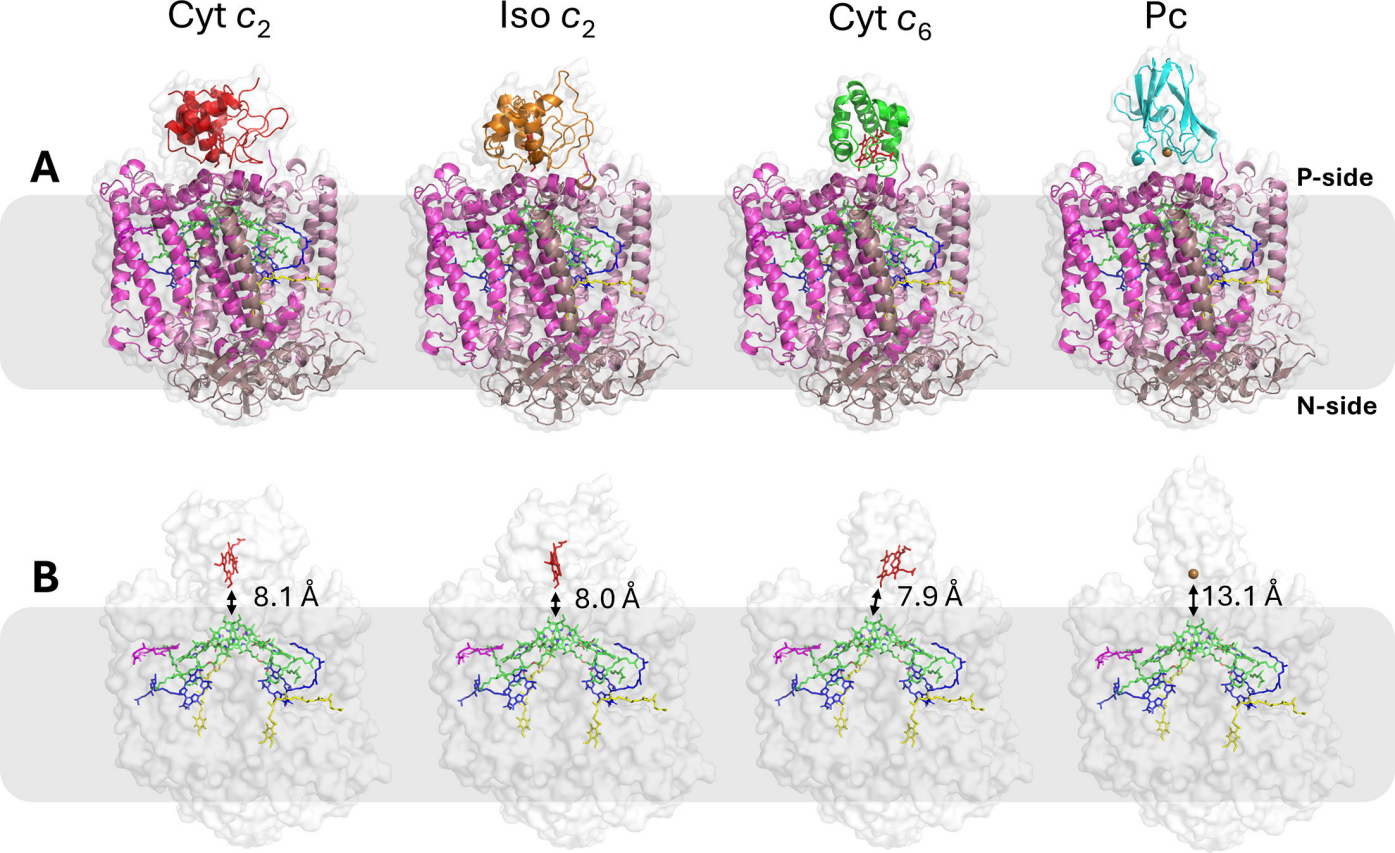
AlphaFold3-predicted complexes of the *Rba. sphaeroides* RC with native and non-native redox carrier proteins. (**A**) Cartoon representations of the RC-L (pink), RC-M (magenta) and RC-H (brown) core subunits bound to cytochrome *c*_2_ (cyt *c*_2_) (red), isocytochrome *c*_2_ (iso *c*_2_) (orange), cytochrome *c*_6_ (cyt *c*_6_) (green) and plastocyanin (Pc) (cyan). (**B**) Transparent space-filling models of the complexes, with cofactors visible including heme (red), copper (orange), BChl (green), bacteriopheophytin (blue) and UQ (yellow). Distances are labelled, with the predicted edge-to-edge distances between the heme *c* in cyt *c*_2_ and the primary pair BChls being only slightly lower than the 8.4 Å value determined by X-ray crystallography [20].

**Figure 3:**
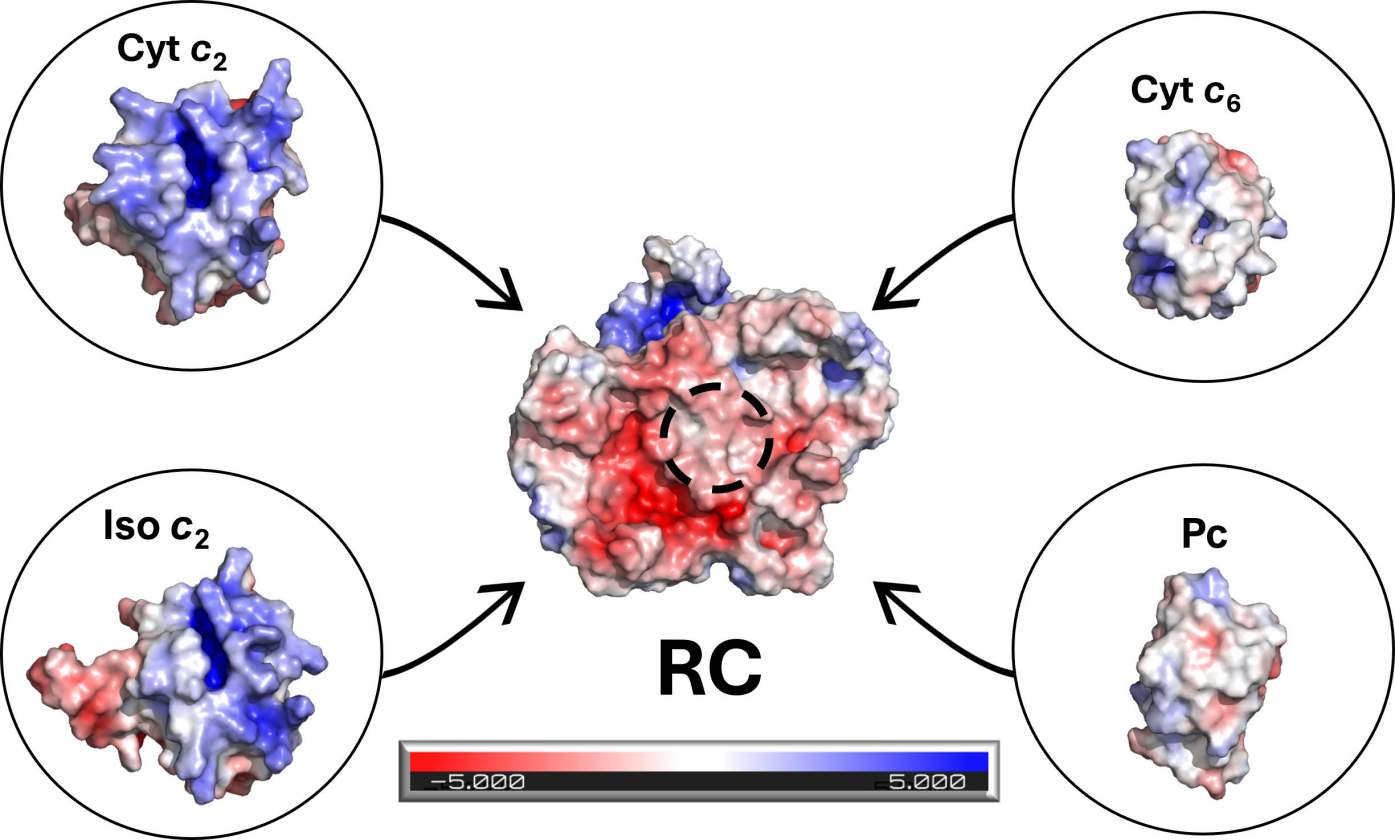
Surface electrostatics of the *Rba. sphaeroides* RC and various soluble electron donors. Surface charges on the binding faces of selected electron transfer proteins in ‘open book’ style, showing the periplasmic face of the RC and the incoming faces of the four electron donors (circled) that dock onto the RC. Structures were generated using AlphaFold2 [48], visualised in PyMol and coloured using the APBS plugin [49], with negative charges in red, positive charges in blue and uncharged patches in white, with colour strength denoting charge density. cyt *c*_2_, cytochrome *c*_2_; cyt *c*_6_, cytochrome *c*_6_; iso *c*_2_, isocytochrome *c*_2_; Pc, plastocyanin; RC, reaction centre.

Despite billions of years of divergent evolution, both cyt *c*_6_ and Pc are predicted by AlphaFold3 [41] to bind the *Rba. sphaeroides* RC in the same position as the co-evolved cyt *c*_2_, with their redox-active cofactors positioned less than 14 Å from the special pair in the predicted co-structures (Figure 2). This was also the case for isocytochrome *c*_2_ (iso *c*_2_), a redundant genomically encoded donor to RC-LH1 which is up-regulated in ‘spd’ suppressor mutants of *Rba. sphaeroides* when the *cycA* gene for cyt *c*_2_ is knocked out [42]. Since the midpoint redox potentials of cyt *c*_6_ (+ 324 mV) and Pc (+ 360 mV) [43] are similar to cyt *c*_2_ (+ 352 mV) [44] and iso *c*_2_ (+ 294 mV) [45], we predicted that all four redox carrier proteins would be capable of reducing P_865_^+^ once bound. The predicted structures also suggest that cyt *c*_6_ and Pc have some affinity for the cyt *c*_2_ binding site on the RC, which is corroborated by protein–protein docking simulations of AlphaFold2-predicted complexes in the HADDOCK 2.4 server [46,47]. In these simulations, the two *Synechocystis* proteins were predicted to make similarly strong vdW interactions with RC-LH1 compared with cyt *c*_2_, but the electrostatic forces were significantly weaker (Supplementary Figure S1), in keeping with their lower surface charge densities (Figure 3). Calculations suggest that the electron transfer rate from Pc is likely to be several orders of magnitude slower than for the cytochromes (Supplementary Table S2), not least because the donor and acceptor cofactors are 13.1 Å apart, the longest of the distances in Figure 2 [20,40,41,43,45,50,51,52].

### Both cyt *c*_6_ and Pc can substitute for cyt *c*_2_ *in vivo*

**Figure 4:**
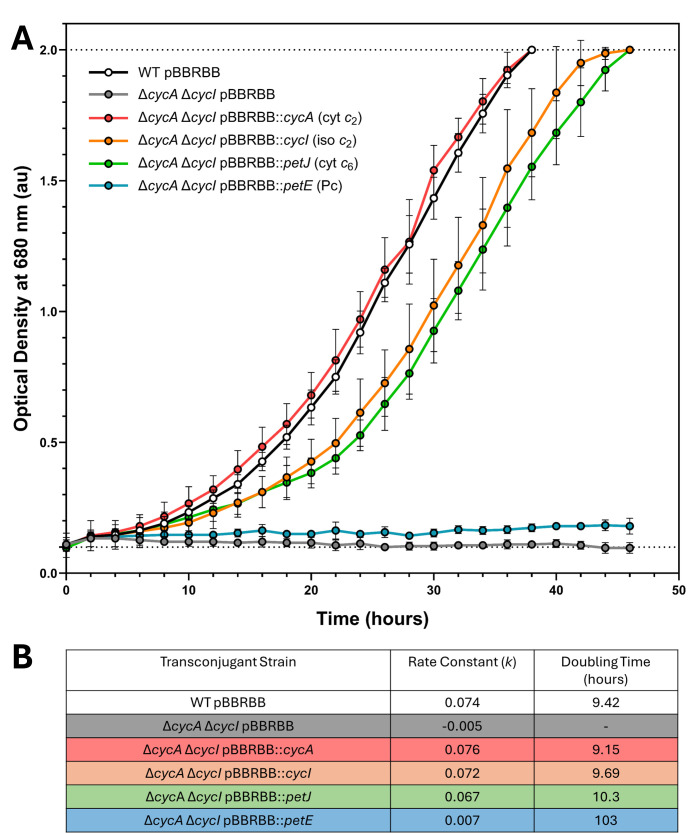
Photoheterotrophic growth curves of *Rba. sphaeroides* strains with native and non-native redox carriers. (**A**) Cultures were grown in triplicate and measured at 2-h intervals. Dotted lines at Y=0.1 and Y=2 represent the starting OD_680_ values and detection limit, respectively. (**B**) Table of growth rate parameters colour coded according to the data in panel (**A**). In panel (**A**) and all subsequent instances, the error bars displayed are +/−1 standard deviation from the mean and *n*=3 unless otherwise stated.

**Figure 5:**
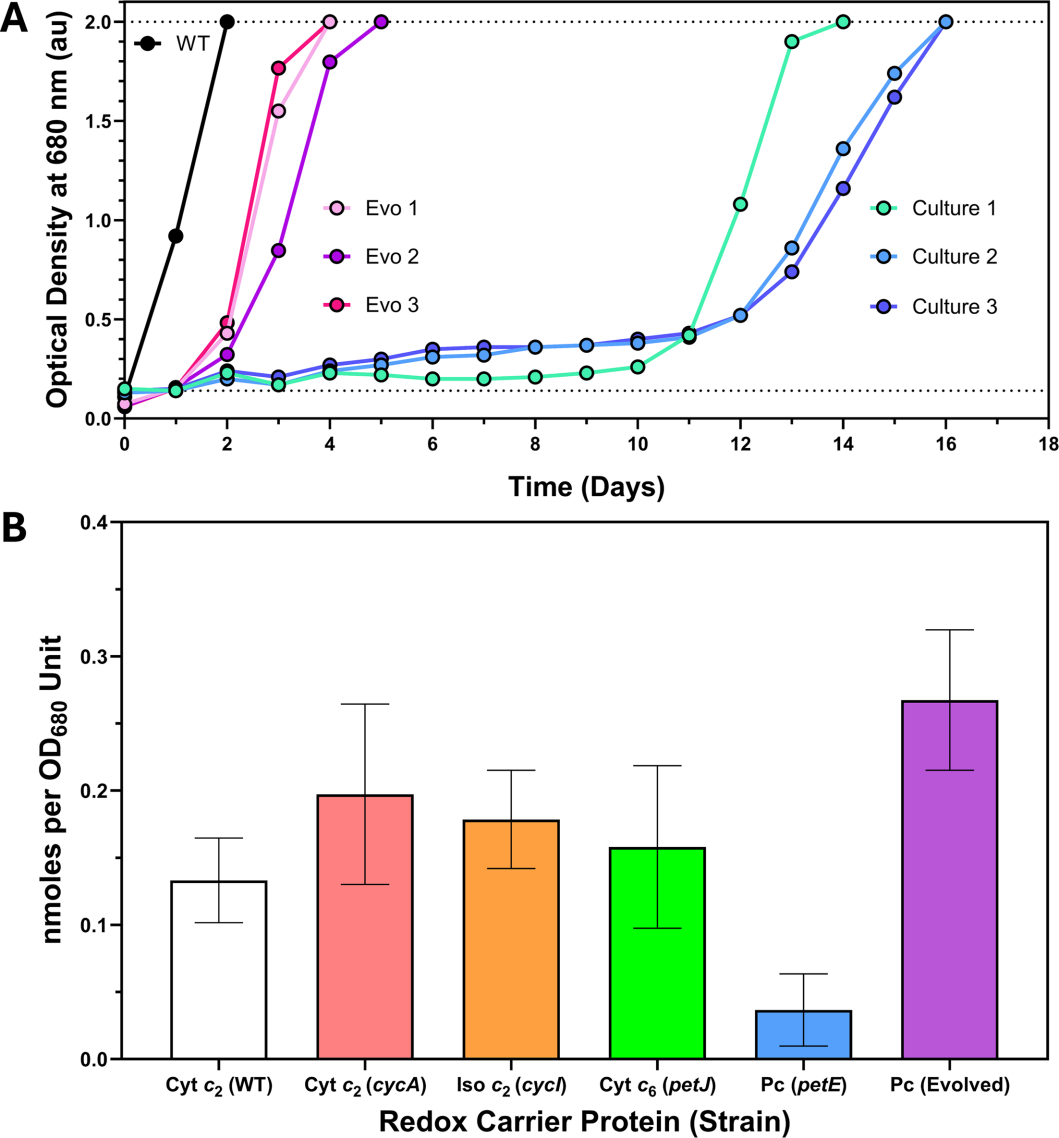
Photoheterotrophic growth curves of *petE* transconjugants and relative periplasmic concentrations of redox carrier proteins in all transconjugant strains. (**A**) Extended photoheterotrophic growth curves comparing WT pBBRBB (positive control, black) to individual *petE* transconjugant cultures (genotype Δ*cycA* Δ*cycI* pBBRBB::*petE*, blue shades). Cells from each *petE* culture were harvested, streaked to single colonies on M22 agar and grown semi-aerobically to make three evolved strains (Evo 1, Evo 2 and Evo 3), with each number corresponding to the culture number from which the cells were isolated. These Evo strains were then grown photoheterotrophically under the same conditions as the original set of growth curves, with the resulting data superimposed onto the 16-day growth curves (pink shades). The average doubling time of the evolved *petE* transconjugants in the second growth experiment was 28.6 h with a rate constant of 0.0243, approximately five times faster than in the first growth experiment. (**B**) The relative amounts of each redox carrier protein that could be extracted from the periplasm of each transconjugant strain using sodium deoxycholate (see Materials and methods). Three biological replicates were conducted for all proteins, except for Pc in the evolved strains (purple), for which *n*=9. cyt *c*_2_, cytochrome *c*_2_; cyt *c*_6_, cytochrome *c*_6_; iso *c*_2_, isocytochrome *c*_2_; Pc, plastocyanin.

To determine how closely computational predictions correspond to *in vivo* behaviour, a photosynthetically incompetent Δ*cycA* Δ*cycI* strain of *Rba. sphaeroides* was constructed which lacked both endogenous redox carrier proteins that can support phototrophic growth [42,53]. Plasmid-borne genes encoding the four different electron donor proteins were transferred to the Δ*cycA* Δ*cycI* strain, generating four transconjugant strains carrying *cycA* (cyt *c*_2_), *cycI* (iso *c*_2_), *petJ* (cyt *c*_6_) or *petE* (Pc). In each transconjugant strain, redox carrier genes were placed under the transcriptional control of a constitutively active promoter, *Ppuf*_843-1200_, in the broad-host replicative vector pBBRBB [54]. Using a consistent starting inoculum of semi-aerobically grown cells, transconjugants were cultured photoheterotrophically under 24 µmol m^-2^ s^-1^ illumination alongside positive wildtype (WT) and negative Δ*cycA* Δ*cycI* control strains carrying empty pBBRBB plasmids. The resulting 48-h growth curves and doubling times presented in Figure 4 demonstrate the very similar growth rates of the WT (empty pBBRBB) control and the *cycA* transconjugant strain (Δ*cycA* Δ*cycI::pBBRBB cycA*), whereas the *cycI* (iso *c*_2_) transconjugant strain (Δ*cycA* Δ*cycI::pBBRBB cycI*) grows slightly more slowly, possibly due to the 40-fold lower affinity iso *c*_2_ has for the RC relative to cyt *c*_2_ [55]. The growth curve for Δ*cycA* Δ*cycI::pBBRBB petJ* shows that cyanobacterial cyt *c*_6_ is sufficiently compatible with RC-LH1, cytochrome *bc*_1_ (cyt *bc*_1_) and the host metabolic machinery to restore a near-WT photoheterotrophic growth rate to the *Rba. sphaeroides* Δ*cycA* Δ*cycI* mutant (Figure 4A). By contrast, the *petE* transconjugants were barely viable, with doubling times over 10-fold longer than the WT (Figure 4B). However, after ~10 days of minimal growth, the photoheterotrophic growth rate of the three *petE* transconjugant cultures suddenly increased five-fold (Figure 5A), possibly arising from a growth-enabling suppressor mutation [42,56,57]. This shorter doubling time was maintained when the suppressor strains were isolated and then cultured photoheterotrophically for a second time, with *petE* transconjugants growing only ~3.1 times more slowly than the WT control and without a lag phase (Figure 5A). Relative quantification of redox carrier proteins in the periplasm using sodium deoxycholate (see Materials and methods) revealed that, unlike cyt *c*_6_, the bioavailability of Pc in *petE* transconjugants is initially very poor, and that the mechanistic basis of improved growth is a dramatic improvement in intracellular Pc levels without any changes to the plasmid sequence or knockout loci (Figure 5B). Despite having a mean periplasmic redox carrier concentration 1.7 times higher than in *petJ* transconjugants (*P*<0.05), the fact that the growth rate of the evolved *petE* transconjugants remained almost three-fold slower is further evidence that RC-LH1 and cyt *bc*_1_ are much more compatible with cyt *c*_6_ than Pc under physiological conditions, assuming that all redox carriers are incorporated equally within chromatophore vesicles. Although different methods were used to quantify cytochrome and Pc levels, Figure 5B shows that the amount of Pc accumulated in the periplasm of the evolved *petE* transconjugant strains was twice that of cyt *c*_2_ in the WT strain (*P*<0.01) and over seven times more than the original *petE* transconjugants before ALE occurred (*P*<0.001).

### RC-LH1 displays very low compatibility with cyt *c*_6_ and Pc in steady-state turnover assays conducted under low ionic strength

**Figure 6:**
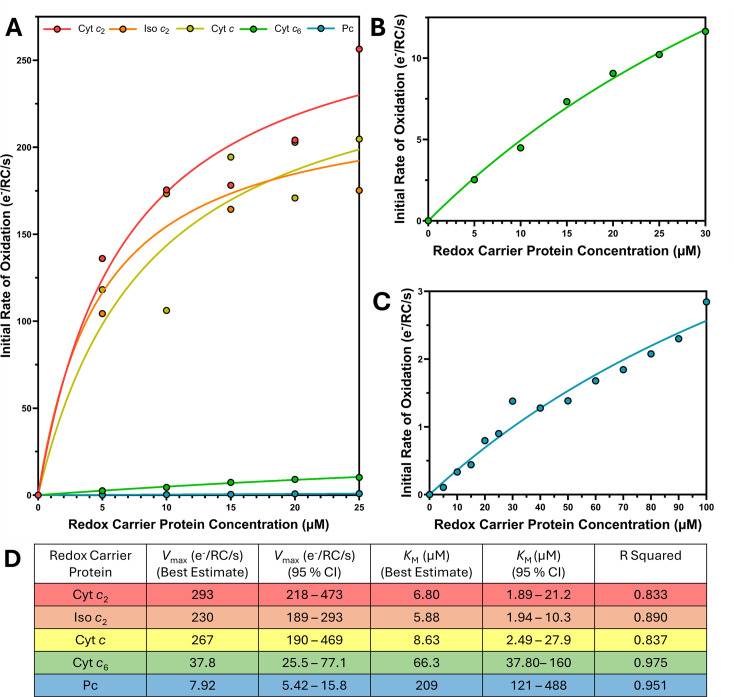
Michaelis–Menten plots, including *V*_max_ and *K*_M_ estimates for oxidation of different redox carriers by RC-LH1 complexes. (**A**) Michaelis–Menten plots of redox carrier oxidation rate at 25°C against the concentration of cyt *c*_2_ (red), iso *c*_2_ (orange), cyt *c* (yellow), cyt *c*_6_ (green) and Pc (blue). (**B**) Expanded Michaelis–Menten plot of redox carrier oxidation rate versus cyt *c*_6_ concentration. (**C**) Expanded Michaelis–Menten plot of redox carrier oxidation rate versus Pc concentration. (**D**) Estimates for *V*_max_ and *K*_M_, along with the 95% confidence intervals for both parameters and the coefficient of determination (R-squared) demonstrating the goodness of fit to the Michaelis–Menten equation used to determine the two parameters in GraphPad Prism 10 (Domatics). 1 mM ascorbate was included in the reaction mixtures of cyt *c*_2_, iso *c*_2_ and cyt *c* to keep each donor reduced before the light-on time. However, this reagent was omitted from reaction mixtures containing cyt *c*_6_ or Pc as these proteins remained reduced during dark adaption and ascorbate addition was found to lower their oxidation rates and amplitudes. cyt *c*, cytochrome *c;* cyt *c*_2_, cytochrome *c*_2_; cyt *c*_6_, cytochrome *c*_6_; iso *c*_2_, isocytochrome *c*_2_; Pc, plastocyanin.

Whilst the behaviour of different redox carriers *in vivo* is a complex function of their affinity for RC-LH1, the cyt *bc*_1_ complex and the host’s wider metabolism, *in vitro* steady-state turnover assays allow direct measurement of the compatibility of the four redox carrier proteins with the RC-LH1 complex. Reaction mixtures of 300 µl were made containing a fixed 0.125 µM concentration of RC-LH1, 50 µM UQ-2 (a soluble analogue of the native UQ-10 substrate with a shorter isoprenoid tail) and a ≥20-fold molar excess of one of the pre-reduced redox carrier proteins, each of which was purified from the periplasm using a novel technique involving sodium deoxycholate (see Materials and methods). In each assay, RC turnover was initiated by continuous illumination with an 810 nm light-emitting diode (LED) and the oxidation of reduced donors monitored by measuring absorbance at their respective redox-sensitive wavelengths. These reactions were conducted in triplicate under the optimum *in vitro* conditions for the RC-LH1-cyt *c*_2_ system (50 mM Tris-HCl at pH 7.5 with 100 mM NaCl and 0.03% (w/v) n-dodecyl-β-D-maltoside (β-DDM)) as determined by separate steady-state turnover experiments (see next section and Supplementary Figure S2). To act as a benchmark for turnover rates, the performance of cytochrome *c* (cyt *c*) from horse heart mitochondria was also measured, since this commercially available protein has been used extensively as an effective *in vitro* analogue of cyt *c*_2_ in historical RC literature despite sharing just 32.7% sequence identity [58,59]. A titration series was conducted for each redox carrier protein and the initial rates of oxidation used to construct Michaelis–Menten plots, from which *V*_max_ and *K*_M_ values could be calculated (Figure 6).

The Michaelis–Menten plots in Figure 6A–C display a clear disparity between the native redox carrier proteins from *Rba. sphaeroides* and their counterparts from *Synechocystis*, with a compatibility order matching the *in vivo* growth curves and the HADDOCK 2.4 predicted affinities. Under the assay conditions, there was little difference in the *V*_max_ and *K*_M_ values for cyt *c*_2_, iso *c*_2_ and cyt *c*, which all lie within error of each other, but the oxidation rates of cyt *c*_6_ and Pc were much slower than the *in vivo* results would suggest (Figure 6D). This indicates that whilst cyt *c*_6_ and Pc can reduce P_865_^+^ fast enough *in vivo* to support photoheterotrophic growth, their intrinsic level of compatibility with the RC-LH1 complex is very low outside the confined chromatophore environment.

### Redox carrier proteins from *Rba. sphaeroides* and *Synechocystis* display opposite salinity trends in their interactions with RC-LH1

Whilst there is a clear oxidation rate hierarchy for the different redox carriers, the conditions used to collect the data in Figure 6 are unlikely to reflect the physiological conditions within chromatophores and may selectively favour the oxidation of the two native redox carriers by RC-LH1. To determine the optimum NaCl concentrations for the oxidation of the five different redox carrier proteins by RC-LH1, steady-state turnover assays were conducted at pH 7.5 with 0.25 µM RC-LH1 and a fixed 80-fold excess of redox carrier protein, along with 50 µM UQ-2, 0.03% (w/v) β-DDM and 1 mM ascorbate for cyt *c*_2_, iso *c*_2_ and cyt *c*, with NaCl added at 25 or 100 mM intervals.

**Figure 7:**
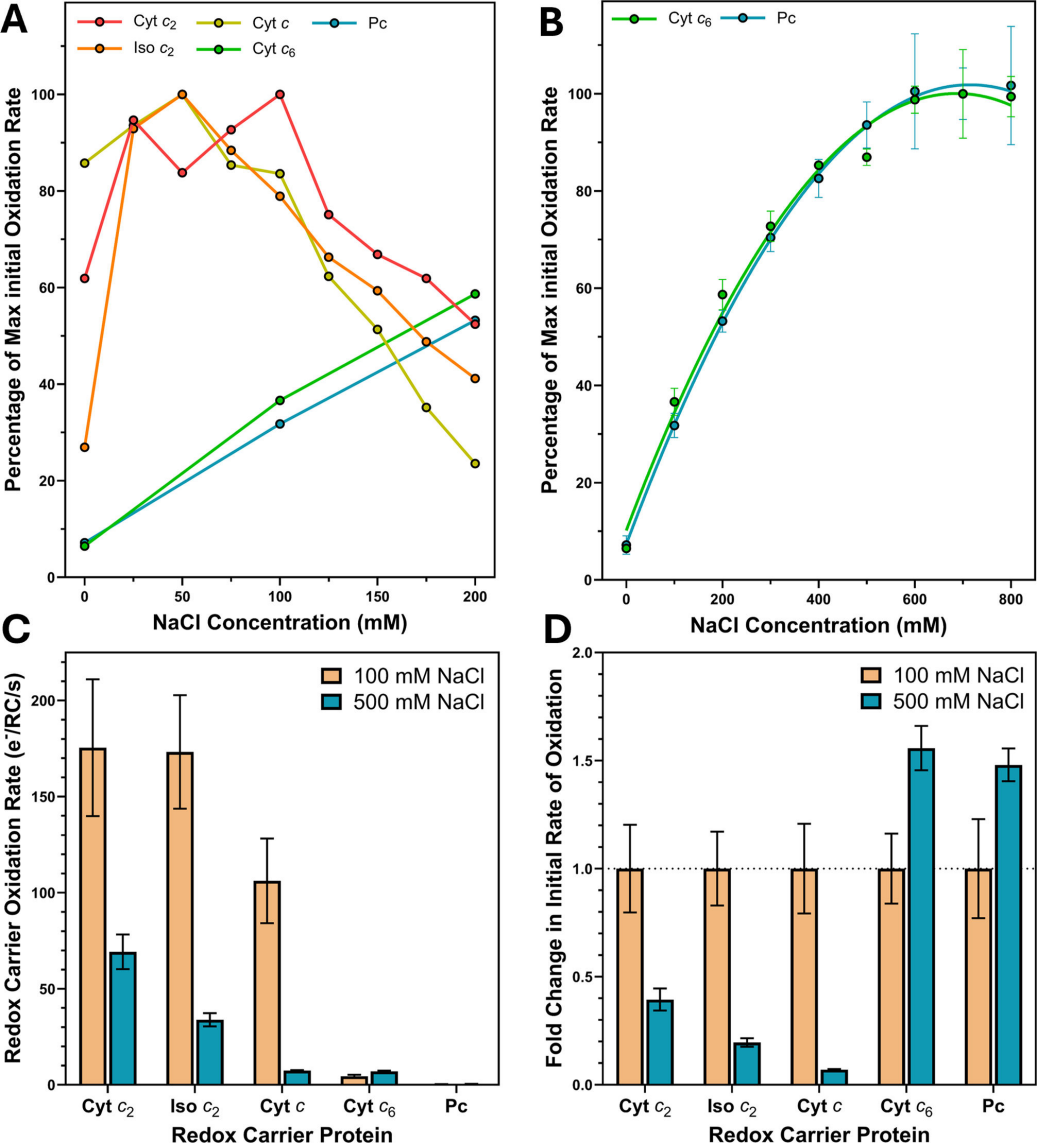
Effect of increasing NaCl concentration on initial rates of redox carrier oxidation by RC-LH1 complexes. Salt dependency of the interactions between RC-LH1 with (**A**) all redox carriers between 0 and 200 mM NaCl and (**B**) Pc and cyt *c*_6_ between 0 and 800 mM NaCl. All reactions were carried out in triplicate and normalised to the maximum rate for each redox carrier, thus the graphs show the percentage change in activity with increasing salinity. Error bars are omitted from panel (**A**) for ease of viewing, with just the mean values plotted for each salt concentration, whilst in panel (**B**), the data are fitted to a centred second-order polynomial (quadratic) function, with R-squared values of 0.98 for both cyt *c*_6_ and Pc. (**C**) Raw initial rates of redox carrier oxidation by RC-LH1 under low salinity (100 mM NaCl, orange) and high salinity (500 mM NaCl, blue). (**D**) Fold change in initial rates of redox carrier oxidation by RC-LH1 from 100 mM (orange) to 500 mM NaCl (blue). All reactions were carried out at 25°C, with each 300 µl reaction mixture containing 0.25 µM RC-LH1, 10 µM redox carrier protein, 50 µM UQ-2, 50 mM Tris-HCl at pH 7.5 and 1 mM ascorbate for cyt *c*_2_, iso *c*_2_ and cyt *c*. (**A**) Raw oxidation rates for the data in panels (**A**) and (**B**) can be found in Supplementary Figure S3. cyt *c*, cytochrome *c;* cyt *c*_2_, cytochrome *c*_2_; cyt *c*_6_, cytochrome *c*_6_; iso *c*_2_, isocytochrome *c*_2_; Pc, plastocyanin.

Plotting oxidation rate against salinity, as shown in Figure 7A and B, reveals decidedly different behavioural trends between the two groups of redox carrier proteins with RC-LH1; whilst the oxidation rates of cyt *c*_2_, iso *c*_2_ and cyt *c* are highest between 25 and 100 mM NaCl, the optimum NaCl concentrations for cyt *c*_6_ and Pc are much higher, lying between 600 and 800 mM. These dramatically increased, and nearly identical, optimum salt concentrations for cyt *c*_6_ and Pc may be a consequence of incompatible electrostatic interactions with RC-LH1 that are screened at high salinities. Amongst the three redox carrier proteins that perform better under lower salinities, it is perhaps unsurprising that the physiological RC-LH1 electron donor, cyt *c*_2_, has both the highest optimum NaCl concentration and is the least affected by salt-screening, with the RC-LH1-cyt *c*_2_ system retaining about 40% of its maximum activity at 500 mM NaCl (Figure 7C). By contrast, the interaction between RC-LH1 and cyt *c* is more easily disrupted by salinity, having lost 90% of its cyt *c* oxidation activity by 500 mM (Figure 7C), with an observed 50 mM optimum NaCl concentration comparable with the 40-mM value found previously for the antenna-free RC-cyt *c* system (Figure 7A) [59]. The decline in iso *c*_2_ oxidation rate is intermediate between cyt *c*_2_ and cyt *c*, with only 20% of maximum activity remaining at 500 mM, revealing a clear order of electrostatic complementarity, where cyt *c*_2_ makes the most specific and high-affinity interactions with RC-LH1. A consequence of this differential salt dependency is that at much higher salt concentrations, the gap in RC-LH1 reduction activity between the native and *Synechocystis* redox carriers is significantly lower (Figure 7C), although the compatibility of Pc remains poor.

### LH1 ring subunits modulate both acceptor and donor side electron transfer activity

**Figure 8:**
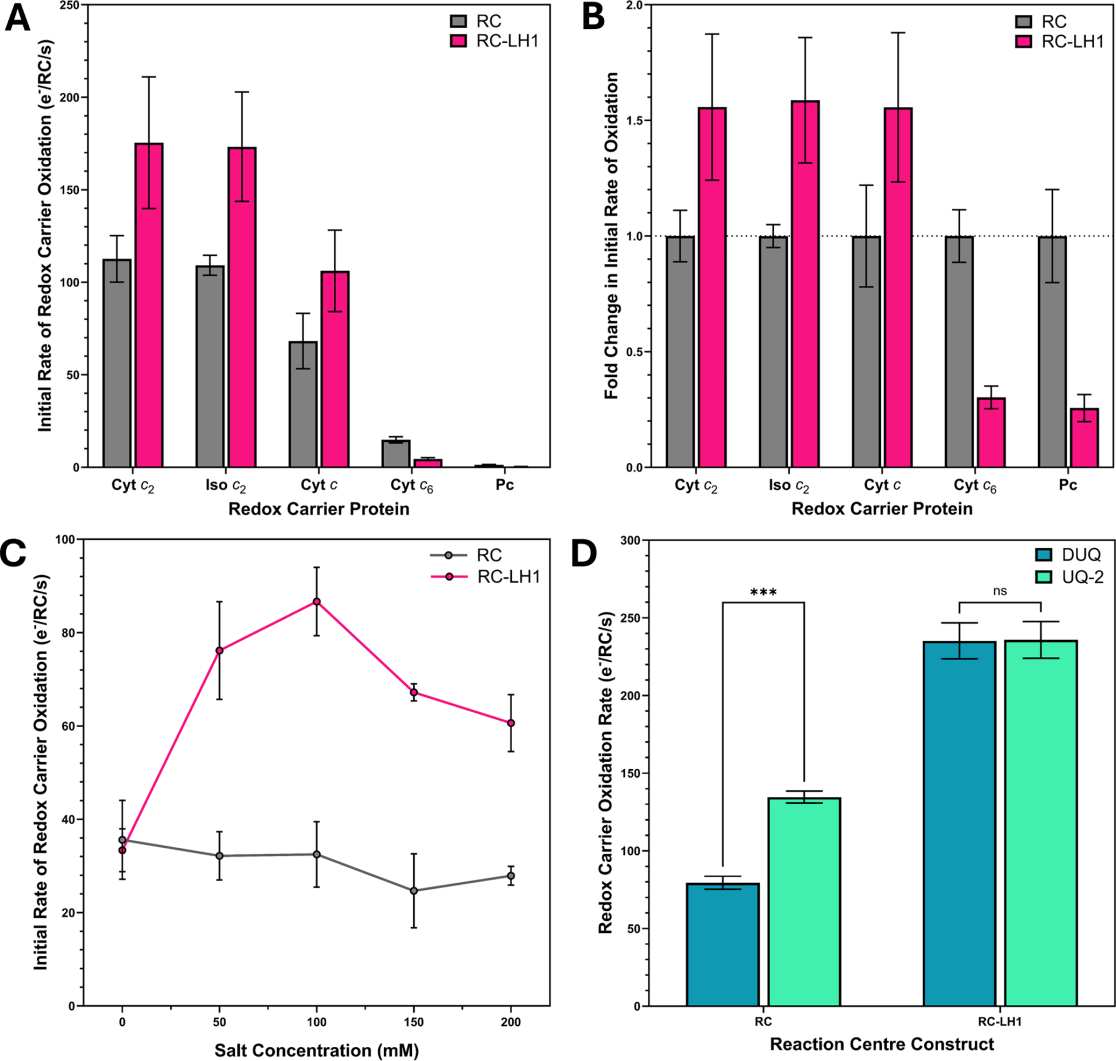
Kinetic behaviour of RC-only and RC-LH1 complexes under different conditions. (**A**) Raw initial rates of redox carrier oxidation during steady-state turnover assays by RC-LH1 (pink) and RC-only complexes (grey). Assays were conducted at 25°C in 50 mM Tris-HCl pH 7.5 and 0.03% (w/v) β-DDM, with 0.125 µM RC or RC-LH1, 10 µM redox carrier, 50 µM UQ-2 and 1 mM sodium D-ascorbate. (**B**) Fold change in RC-LH1 initial oxidation rates compared with RC-only for each redox carrier protein, from the dataset plotted in (**A**). (**C**) Initial rates of cyt *c*_2_ oxidation by RC-LH1 (pink) and RC-only complexes (grey) in response to increasing [NaCl], with conditions of 0.5 µM RC/RC-LH1, 10 µM cyt *c*_2_ and 0.03% (w/v) β-DDM in 50 mM Tris-HCl pH 8, along with 1 mM ascorbate for reaction mixtures containing RC-LH1 but not RC-only complexes. (**D**) Bar chart comparison of initial rates of cyt *c*_2_ oxidation by RC-LH1 and RC-only complexes with two different quinone analogues, decylubiquinone (DUQ, blue) and UQ-2 (cyan). Reactions were carried out with 0.5 µM RC/RC-LH1, 11 µM cyt *c*_2_, 0.5 mM ascorbate and 50 µM DUQ/UQ-2, with a *P* value of <0.001 (***) for the RC-only complex with the two different analogues, calculated from an unpaired *t*-test using the Holm–Šídák method to account for multiple comparisons. cyt *c*, cytochrome *c*; cyt *c*_2_, cytochrome *c*_2_; cyt *c*_6_, cytochrome *c*_6_; iso *c*_2_, isocytochrome *c*_2_; Pc, plastocyanin.

Since the first RC purification by Reed and Clayton in 1968 [60], it has been a common practice to solubilise *Rba. sphaeroides* membranes in detergents such as Triton X-100 or lauryldimethylamine oxide (LDAO) which removes LH1-associated subunits (mainly LH1 α, β, as well as X, Y and Z), leaving behind the more experimentally accessible RC-only complex. In steady-state turnover assays, this truncated complex, which consists of just the three core RC L, M and H subunits, behaves very differently to the physiological RC-LH1 system under its optimal conditions (50 mM Tris at pH 7.5, 100 mM NaCl). Not only is the cyt *c*_2_ oxidation rate by the RC-only complex more than 30% slower than that by RC-LH1 (Figure 8A and B), but it also appears to be salt concentration independent under the conditions of this steady-state assay (Figure 8C). Since only the solvent-exposed cyt *c*_2_-binding site (donor side) of the complex is affected by salt, this result suggests that the activity of the RC-only complex is held back by Q_B_ turnover, making it acceptor side limited. This theory is corroborated by an observed increase in cyt *c*_2_ oxidation rate by RC-only complexes when decylubiquinone (DUQ) was exchanged for UQ-2, a UQ analogue with a more physiological tail composed of two isoprene units rather than a saturated decane chain, whilst no difference in behaviour was observed between these two analogues with RC-LH1 (Figure 8D). This phenotype was found to be highly robust and repeatable, being observed consistently across many technical and biological repeats, including when RC-only complexes were purified from LH1-minus Δ*pufBA* strains or directly from photoheterotrophically grown RC-LH1 complexes containing spheroidene by LDAO treatment (data not shown). Follow-up steady-state turnover experiments also confirmed that this difference in quinone analogue sensitivity between the two forms of the RC complex cannot be explained by the additional light-harvesting capacity of the LH1 ring, since under the conditions of the assay, the RC-only complex is already light-saturated (Supplementary Figure S4).

Along with responding differently to salinity, cyt *c*_6_ and Pc also display opposite trends to cyt *c*_2_ with the truncated RC-only complex (Figure 8A and B). Whilst the initial oxidation rates of cyt *c*_2_, iso *c*_2_ and cyt *c* are all improved ~1.5-fold by switching from the RC-only to the RC-LH1 version of the complex, the opposite is true for cyt *c*_6_ and Pc, whose oxidation rates drop three-fold. This suggests that there is a fundamental incompatibility between the *Synechocystis* redox carrier proteins and the LH1-associated subunits that surround the RC core complex. Collectively, these subunits appear to not only confer higher acceptor side activity by improving quinone reduction but also seem to impart greater donor side selectivity by hindering the binding of non-cognate proteins that lack the correct properties. The LH1 ring may also promote cyt *c*_2_ binding through electrostatic steering, as suggested in our recent single molecule force spectroscopy study [28], which could also contribute to the observed difference in cyt *c*_2_ oxidation rates between RC-only and RC-LH1 complexes.

## Discussion

The data presented in the present study demonstrate that despite billions of years of divergent evolution, the *Rba. sphaeroides* RC-LH1 complex displays a surprisingly high level of *in vivo* compatibility with the cyanobacterial cyt *c*_6_ and Pc redox carriers from *Synechocystis* sp. PCC 6803, which usually reduce PSI. Although both *Synechocystis* proteins can shuttle electrons between the RC-LH1 and cyt *bc*_1_ complexes efficiently enough to support photoheterotrophic growth *in vivo*, *in vitro* and *in silico* data paint a more complex picture of compatibility compared with the two native redox carrier proteins in *Rba. sphaeroides*. Computational modelling predictions, photoheterotrophic growth rates and steady-state turnover assays all converge on a predicted RC-LH1 compatibility order where cyt *c*_2_ is the best electron donor to P_865_, followed by iso *c*_2_, then cyt *c*_6_ and finally Pc, but different methods give different relative estimates due to fundamental differences in behaviour between these redox carrier proteins. Whilst cyt *c*_2_ and iso *c*_2_ were oxidised fastest by RC-LH1 at low salinities (50–100 mM NaCl), the highest turnover rates for cyt *c*_6_ and Pc were achieved with the RC-only complex and high salinities (600–750 mM). A third non-native redox carrier, cyt *c* from *Equus caballus*, showed the same trends as the *Rba. sphaeroides* redox carriers, but its performance compared with the native *Rba. sphaeroides* cyt *c*_2_ fell away sharply with increased salinity. Taking all these data together, several key differences emerge between the RC-LH1-cyt *c*_2_ and PSI-cyt c_6_/Pc systems, as well as some unexpected properties of the RC-LH1 complex. Although Pc is intrinsically much less compatible with RC-LH1, likely because it hails from a different family of proteins, the identical behavioural trends exhibited by cyt *c*_6_ and Pc are in keeping with their isofunctional roles in thylakoid electron transfer [61,62]. Regardless of the nature of their interaction, it may be that any reduced electron carrier protein with a suitable redox potential can reduce P_865_^+^ if its redox-active cofactor can be positioned within 14 Å of the special pair.

The rate at which different redox carrier proteins can reduce the RC-LH1 complex in the steady state is a function of three countervailing processes, binding (*k*_on_), electron transfer (*k*_ET_) and unbinding (*k*_off_), all of which require a range of different parameters to be maintained within a narrow range. Protein–protein docking in AlphaFold3 and HADDOCK 2.4 suggests that both cyt *c*_6_ and Pc bind RC-LH1 in sufficient proximity and in the correct orientation to support productive electron transfer (Figure 2) (Supplementary Figure S1), but their different surface properties are a barrier for binding to the RC (Figure 3). Whilst both long-range electrostatic steering and short-range hydrophobic interactions are important for binding both PSI and RC-LH1, the binding partners in *Rba. sphaeroides* have a much greater electrostatic force component, whilst cyt *c*_6_ and Pc have much lower surface charge densities (Figure 3). As a result of these differences, it may be that the PSI cyt *c*_6_/Pc binding interface is intrinsically weaker than RC-LH1-cyt *c*_2_/iso *c*_2_, especially since the experimentally determined *K*_D_ for plant PSI-Pc [63] is 70 times greater than RC-cyt *c*_2_ [64].

**Figure 9:**
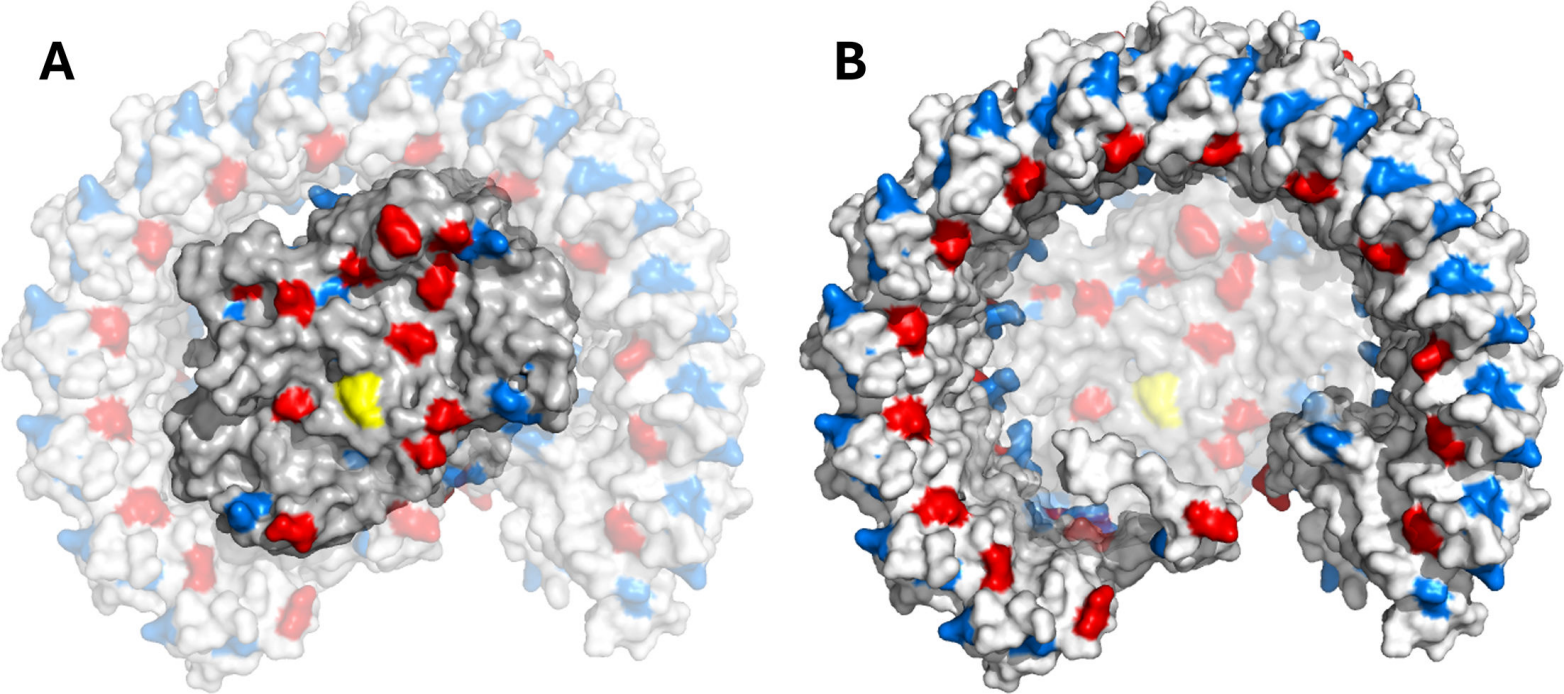
Comparison of surface electrostatics on the periplasmic faces of the RC and LH1 components of the RC-LH1 complex. (**A**) Surface representation of the periplasmic faces of the RC-L and RC-M subunits with other subunits set to 65% transparency, whilst (**B**) shows the periplasmic face of the rest of the complex, including all ring subunits (α, β, X, Y and Z chains), with RC-L and RC-M set to 65% transparency. In both (**A**) and (**B**), amino acids with negatively charged side chains are coloured red, with positively charged residues in blue and Tyr-L162 shown in yellow to mark the site of electron transfer. Structure shown in this figure is the RC-LH1 monomer complex from [16], PDB 7PIL.

Very low oxidation rates were measured for cyt *c*_6_ and Pc with RC-LH1 in the 50-150 mM NaCl range that favours the native cyt *c*_2_ electron donor, but were greatly improved at high salinities in the 500-800 mM range (Figure 7). The binding faces of cyt *c*_6_ and Pc are not highly charged, unlike cyt *c*_2_ (Figure 3), but the highly negatively charged surface of the RC-LH1 complex (Figure 9A) could present a significant barrier to forming initial encounter complexes with cyt *c*_6_ and Pc, especially if unproductive electrostatic interactions are occurring between the RC-LH1 complex and the non-binding faces of cyt *c*_6_ and Pc. We suggest that high salinities mask these incompatibilities and this could also explain why cyt *c*_6_ and Pc oxidation rates improve when the highly charged LH1 ring is removed from the complex.

Taken together, the differential effects of salt screening and subunit composition on the redox carrier proteins also suggest that the subunits that surround the RC core complex are required not only for light harvesting but also for maximising the activity and selectivity of electron donors under optimal conditions. If the surface charges on each LH1 αβ pair can be unfavourable for the binding of cyt *c*_6_ and Pc, then it is likely that the opposite can be true for the native RC-LH1 electron donor, with molecular dynamics data suggesting that the LH1 ring has a degree of affinity for cyt *c*_2_ [28]. It is, therefore, possible that LH1 αβ pair electrostatics are partially responsible for the observed rate difference in cyt *c*_2_ oxidation between RC-only and RC-LH1 complexes, with possible roles for surface charges in recruitment, orientation, and entrance and exit pathways for redox carrier proteins (Figure 9B). However, this difference can also be explained by an acceptor side limitation in the truncated three-subunit RC-only complex, which is supported by the sensitivity of its turnover rate to different quinone analogues. The way that RC-LH1 complexes are donor-side limited under the same conditions and do not respond to quinone analogue changes suggests the LH1 α and β polypeptides that surround the RC, along with the X, Y and Z subunits, act in concert to increase acceptor side quinone turnover. The mechanism by which these subunits enhance acceptor side turnover could involve providing efficient entrance and exit channels, providing a ‘conveyor belt’ pathway for quinones to rapidly enter the Q_B_ binding site and leave as soon as they are doubly reduced [65,66]. Additional low-affinity UQ binding sites, which might act as a ‘waiting room’ to increase the local concentration of incoming quinones, have already been identified in cryo-EM structures of the *Rhodopseudomonas palustris* RC-LH1 complex and involve π-stacking interactions between tryptophan residues and quinone head groups [65,67], whilst π-stacking and hydrogen bonding with side chains on LH1 αβ subunits have also been implicated in quinol exit pathways [66]. In the *Rba. sphaeroides* RC-LH1 complex, accessory entrance and exit sites might be found in the transmembrane helices of the αβ pairs, or might even be associated with the X, Y and Z subunits which are already known to maintain open quinone channels to the membrane, completing a ‘highway’ for quinone diffusion between RC-LH1 and cyt *bc*_1_ [53,67–71].

Ultimately, however, under almost all light levels, the activity of the RC-LH1 complex is not limiting for photosynthetic growth; the slowest step in the cyclic purple bacterial electron transport chain is widely acknowledged to be quinol oxidation by the cyt *bc*_1_ complex instead [72–74]. This means that a non-native redox carrier protein substituting for cyt *c*_2_ would not limit photosynthetic growth rate if it oxidised cyt *bc*_1_ and reduced RC-LH1 more rapidly than the cyt *bc*_1_ complex can oxidise ubiquinol. For *petJ* and *petE* transconjugant strains growing photoheterotrophically (Figure 4), a slower rate of RC-LH1 re-reduction could be tolerated with only a slight increase in doubling time, masking the true difference in compatibility between the native redox carrier proteins and their counterparts from *Synechocystis*. This could explain why, following ALE of *petE* transconjugants by a currently unknown mechanism, the compatibility of cyt *c*_6_ and Pc unusually appears higher *in vivo* than *in vitro*, rather than the other way around.

The differences between *in vivo* and *in vitro* data summarised in Table 1 can also be reconciled in the context of the conditions of the chromatophore lumen, which will almost certainly have a pH, viscosity and salinity that differ from laboratory conditions whilst also containing different types of salts and solutes, plus a lipid bilayer. The conditions inside a chromatophore vesicle cannot be replicated *in vitro* and in this tiny compartment, which has a typical internal diameter of just 45 nm [75], *K*_D_ and *K*_M_ values are rendered irrelevant by the effective redox carrier concentration within the chromatophore lumen, which for cyt *c*_2_ is estimated to be 600 μM [73], a value of 3 orders of magnitude greater than the *K*_D_ value of 0.3 µM for the RC-cyt *c*_2_ system [64]. The periplasmic concentrations for cyt *c_6_* and Pc in the ALE strains are comparable to that for cyt *c*_2_ (Figure 5B) and, therefore, still exceed the measured *K*_M_ values of 66 and 209 µM, respectively. Thus, *in vivo* compartmentalisation of reactants can overcome apparent *in vitro* limitations, and in this case, excess amounts of cyt *c*_6_ and Pc are already capable of productively shuttling electrons between RC-LH1 and cyt *bc*_1_, raising the prospects that synthetic biology can bring together photosynthetic proteins that evolution has long since separated.

**Table 1:** Summary of experiments conducted with host and *Synechocystis* redox carrier proteins

	Cyt *c*_2_	Iso *c*_2_	Cyt *c*_6_	Pc
Edge-to-edge distance (Å)	8.1	8.0	7.9	13.1
HADDOCK Score	−183	−144	−104	−91.9
Predicted *k*_et_ (s^−1^)	1.38×10^9^	7.53×10^9^	3.23×10^9^	2.77×10^5^
Doubling time (hours)	9.15	9.69	10.3	36.1
*V*_max_ (e^−^/RC/s)	293	230	37.8	7.92
*K*_M_ (µM)	6.80	5.88	66.3	209
Optimum [NaCl] (mM)	100	50	700	700

Cyt *c*_2_, cytochrome *c*_2_. Cyt *c*_6_, cytochrome *c*_6_. Iso *c_2_*, isocytochrome *c*_2_. Pc, plastocyanin.

## Materials & methods

### Generation of strains and plasmids

The work described in the present study was carried out in several strains of *Rba. sphaeroides* 2.4.1, along with *E. coli* strains for molecular cloning and conjugative transfer of plasmids, all of which are listed in Table 2. For complementation studies and protein production, an unmarked genomic deletion strain lacking the endogenous photosynthetic redox carrier genes (genotype Δ*cycA* Δ*cycI*) was generated using the pK18mob*sacB* system as previously described [53]. The DX13 and E9 strains lacking various RC-LH1 and LH2 genes were generated by sequential rounds of pK18mob*sacB* mutagenesis following the protocol detailed in [79]. Unmodified genes encoding cyt *c*_2_ (*cycA*), iso *c*_2_ (*cycI*), cyt *c*_6_ (*petJ*) and Pc (*petE*) were PCR-amplified from their host organism using primers listed in Supplementary Table S1, digested with BglII and XhoI/SalI (Thermo Fisher Scientific), then ligated into the broad-host range vector pBBRBBB-*Ppuf*_843-1200_ [54]. Following sequence verification (Eurofins Genomics), the resulting plasmids were transformed into *E. coli* S17-1 and conjugated into the Δ*cycA* Δ*cycI* strain of *Rba. sphaeroides*, using 30 µg ml^-1^ kanamycin to select transconjugants. A similar strategy was employed for the RC subunit-encoding *pufLM* genes, with PCR fragments being cloned into the same vector at the BglII/SpeI restriction sites, and conjugation of the ligated plasmid into the E9 (Δ*puc1BA* Δ*pufBALM*) strain. A *petE*-StrepII-tag insert was also made by PCR from *Synechocystis* sp. PCC 6803 cells are cloned into a pET28a vector using NcoI and XhoI restriction sites, then transformed into the *E. coli* BL21(DE3) strain (Promega) for Strep-tagged Pc production.

**Table 2:** Bacterial strains used in the present study

Species/strain	Genotype	Properties	Source/reference
***Rhodobacter sphaeroides*** **2.4.1**			
WT	N/A	WT strain	S. Kaplan (University of Texas)
Δ*cycA* Δ*cycI*	Δ*cycA* Δ*cycI*	Unmarked deletion of *cycA* (RSP_0296) and *cycI* (RSP_2577); does not produce cyt *c*_2_ (*cycA*) or iso *c*_2_ (*cycI*)	[53]
DX13	Δ*puc1BA* Δ*puc2BA* PufX R49L R53L	Unmarked deletion strain of LH2 genes, *puc1BA* (RSP_0314–0315) and *puc2BA* (RSP_1556–1557). Replacement of arginine residues in PufX responsible for dimerisation with leucine so the strain exclusively makes RC-LH1 monomers	This study S. Kaplan (University of Texas) (Δ*puc1BA*) [76](Δ*puc2BA*) [77](PufX R49L R53L)
E9	Δ*puc1BA* Δ*pufBALM*	Unmarked deletion strain of LH2 genes, *puc1BA* (RSP_0314–0315), along with *pufBALM* (RSP_6108, RSP_0258–0256) encoding the RC-LH1 L, M, α and β subunits	This study S. Kaplan (University of Texas) (Δ*puc1BA*)
***Escherichia coli***			
Bl21(DE3)	F–*omp*T *hsd*SB (r_B_^–^, m_B_^–^) *gal dcm* (DE3)	Protein production strain	Promega
JM109	*end*A1, *rec*A1, *gyr*A96, *thi*, *hsd*R17 (r_k_^–^, m_k_^+^), *rel*A1, *sup*E44, Δ(*lac-proAB*), [F´ *tra*D36, *pro*AB, *laqI* ^q^ZΔM15]	Cloning strain	Promega
S17-1	RP4-2 (Tc::Mu, Nm::Tm7) integrated into the chromosome *Tp* ^R^ *Sm* ^R^ *rec*A, *thi*, *pro*, *hsd*M^+^	Conjugative transfer of plasmids to *Rba. sphaeroides*	[78]
***Synechocystis *** **sp. PCC 6803**			
WT	N/A	Glucose-tolerant WT strain	R. Sobotka (Algatech, Třeboň)

WT, wildtype.

### Semi-aerobic growth of *Rba. sphaeroides* cells

*Rba. sphaeroides* cells were cultured in M22+ medium [80]. Kanamycin (30 µg ml^-1^) was added to maintain plasmids where necessary. Three different types of flat-bottomed glassware were used to make liquid cultures. In the first liquid culture step, universal tubes containing 10 ml of medium (about 25% full) were inoculated with single colonies from M22 agar plates and grown at 30°C with shaking at 150 rpm until pigmented (~24–48 h). A single 10 ml culture was then used to inoculate either 80 ml medium in a 125-ml Erlenmeyer flask and grown overnight, or 1.6 L medium in a 2-L conical flask and grown for 72 h. The universal tubes were sealed with a screw top cap; the other cultures were kept sterile with a cotton wool bung topped with two layers of aluminium foil.

### Photoheterotrophic growth of *Rba. sphaeroides* cells

Anoxic conditions promoting photoheterotrophic growth were achieved by fully filling and stoppering glassware with M22+medium. Larger cultures for RC-LH1 purification were grown in 1-l Roux bottles stoppered with a rubber bung, whilst for growth curves, narrow ~18-ml glass Hungate tubes were inoculated with cells from semi-aerobic 80-ml cultures, using the OD_680_ value to estimate culture turbidity and calculate the amount of inoculum needed to ensure a consistent starting cell density of ~0.1. Cultures were illuminated by a 70-W Halogen Classic Bulbs (Phillips) usually at 25 µmol photonsm^-2^ s^-1^ and agitated with magnetic stirrers until reaching stationary phase, which corresponds to an approximate OD_680_ of 3. Growth curve culture turbidity at 680 nm was measured with a C07500 Colorwave colorimeter (WPA). The doubling times of photoheterotrophic cultures were calculated by non-linear fitting in GraphPad Prism 10 using the exponential (Malthusian) growth function.

### Purification of intracytoplasmic membranes

All RC-LH1 complexes were purified as monomers from DX13 cells (Δ*puc1BA* Δ*puc2BA* PufX R49L R53L) grown photoheterotrophically in a 1-L Roux culture bottle as described above. Cells were harvested at 4200*×**g*** for 30 min at 4°C, and the resulting pellets were resuspended in 50 mM Tris-HCl pH 8 supplemented with an EDTA-free protease inhibitor tablet (Merck) to a total volume of 30 ml. Resuspended cells were incubated at room temperature with lysozyme for 20 min, before DNase I was added and the cells were placed on ice. Cells were lysed by two passages through a chilled French press at 124 MPa, and the lysate was clarified by centrifugation at 27,000× ***g*** for 20 min at 4°C to remove cell debris. Intracytoplasmic membranes (ICM) were prepared by layering clarified supernatant on top of a 15/40% (w/v) discontinuous sucrose gradient, then centrifuged at 84,500× ***g*** for 10 h at 4°C. The pigmented band of ICM formed at the interface was harvested with a serological pipette and centrifuged again at 185,500× ***g*** for 2 h at 4°C.

### Purification of RC-LH1 complexes

Pelleted ICM were resuspended in 50 mM Tris-HCl pH 8 and homogenised before being solubilised in 2% (w/v) β-DDM. RC-LH1 complexes were purified by a round of anion exchange on a 150 ml diethylaminoethanol (DEAE) Sepharose column at 5 ml min^-1^, using a gradient of 200–300 mM NaCl in 20 mM Tris-HCl pH 8 with 0.03% (w/v) β-DDM. After spin concentration using 100-kDa molecular weight cut-off (MWCO) concentrators (Thermo Fisher), gel filtration chromatography was carried out on a HiLoad® 16/600 Superdex® 200 pg column (Cytiva) in 50 mM Tris-HCl pH 8 with 200 mM NaCl and 0.03% (w/v) β-DDM at a flow rate of 0.5 ml min^-1^.

### Purification of RC-only complexes

RC-only complexes containing spheroidenone were purified from E9 pBBRBB-*Ppuf*_843-1200_ *:: pufLM* transconjugants grown semi-aerobically as described earlier. Cells were pelleted and lysed as described earlier but, rather than preparing ICM, membranes were harvested from clarified lysate by ultracentrifugation at 185,500*×**g*** for 2 h at 4°C in a type 45 Ti ultracentrifuge rotor (Beckman) and transferred to a small Duran bottle containing 100 ml 50 mM Tris-HCl pH 8. These membranes were resuspended and solubilised at room temperature by stirring in the presence of 1% (v/v) LDAO, deliberately harsh conditions to remove other membrane proteins in the mixture. After loading onto a homemade 150 ml DEAE Sepharose column, RCs were purified by anion exchange in 20 mM Tris-HCl pH 8 with 0.1% (v/v) LDAO using a 160–240 mM NaCl gradient, and a 5 ml min^-1^ flow rate on an ÄKTA Prime FPLC (Cytiva). Purified eluate was spin concentrated to <0.5 ml with 50-kDa MWCO centrifugal concentrators (Sartorius) and then purified further by gel filtration at 0.5 ml min^-1^ on a HiLoad® 16/600 Superdex® 200 pg column (Cytiva) into 50 mM Tris-HCl pH 7.5, with 100 mM NaCl and 0.03% (w/v) β-DDM.

RCs containing spheroidene were purified from RC-LH1 complexes (see above) from anaerobic, phototrophically grown strains, by harnessing the sensitivity of LH1 αβ subunit pairs to LDAO treatment. RC-LH1 complexes were buffer-exchanged into 50 mM Tris-HCl buffer at pH 8 with 0.1% (v/v) LDAO and loaded onto a 5ml Q Sepharose column (Cytiva). Bound complexes were then washed with at least 10 column volumes of the same buffer with 4% (v/v) LDAO, before RC-only complexes were eluted using 20 mM Tris-HCl at pH 8 with 0.1% (v/v) LDAO and a 200–400 mM NaCl gradient at a flow rate of 5 ml min^-1^.

### Purification of *c*-type cytochromes from *Rba. sphaeroides*

All three untagged cytochromes (cyt *c*_2_, iso *c*_2_ and cyt *c*_6_) were purified from their respective transconjugant strains by first performing a novel deoxycholate-based fractionation technique, which efficiently separated the contents of the periplasm from the bulk of the cellular proteome. Although the downstream purification steps varied between the four proteins, the periplasmic extraction process was the same each time. The 1.6-L semi-aerobic cultures were pelleted at 4200× ***g*** for 30 min at 4°C and resuspended in ~20 ml periplasmic extraction buffer to a total volume of 40 ml, supplemented with EDTA-free protease inhibitor (Merck). This buffer, which was carefully designed to avoid whole cell lysis and deoxycholate hydrogel formation, contained 100 mM HEPES at pH 8 for the two *Rba. sphaeroides* proteins or 100 mM CHES at pH 9 for cyt *c*_6_, along with 500 mM sucrose and 50 mM NaCl as osmotic stabilisers. 0.8 g solid sodium deoxycholate was added to each cell suspension to make a 2% (w/v) solution, which was then incubated in the dark at 4°C with mild agitation. After an hour, spheroplasts were pelleted at 35,000*×**g*** for 30 min at 4°C, and the supernatant, which contains the contents of the periplasm, was taken forward to the next step (see below for specific details for each protein). Once purified, each redox carrier protein was concentrated to 2.5 ml in 3-kDa MWCO spin concentrators (Thermo Fisher Scientific), reduced with sodium dithionite and exchanged into the desired buffer using PD-10 desalting columns (Cytiva) according to the manufacturer’s instructions.

### Purification of untagged cyt *c*_2_

Harnessing the propensity of divalent cations to precipitate bile acids, deoxycholate was removed from the periplasmic fraction by the addition of 6.25 ml 5× deoxycholate precipitation solution (1 M ammonium acetate at pH 5 and 250 mM MgSO_4_), mixing by inverting the tube and centrifugation at 35,000× ***g*** for 30 min at 4°C. The resulting supernatant was then passed through a homemade 30-ml SP Sepharose cation exchange column equilibrated in 50 mM ammonium acetate buffer at pH 5. Any red-coloured eluate was collected, passed through a 0.22-µm filter, diluted 2.5-fold in 50 mM ammonium acetate at pH 5 and clarified by centrifugation at 15,000× ***g*** for 10 min at 4°C, whilst the column was washed with successive column volumes of 1 M NaCl, 1 M NaOH and 100% ethanol. The SP Sepharose column was then re-equilibrated in 50 mM ammonium acetate at pH 5, and the clarified coloured eluate loaded onto the column for a second time, resulting in binding of the target protein. Pure cyt *c*_2_ was eluted over a gradient of 60–160 mM NaCl at 5 ml min^-1^ (Supplementary Figure S5).

### Purification of untagged iso *c*_2_

With its similar electrostatic properties, untagged iso *c*_2_ was purified using the same column and buffer system used for cyt *c*_2_. Following an identical deoxycholate precipitation step and centrifugation, clarified iso *c*_2_ supernatant was passed through a 0.22-µm filter (Sartorius), diluted ten-fold with 50 mM ammonium acetate buffer at pH 5 and loaded directly onto the column. Cation exchange was performed to elute pure iso *c*_2_ over a 150–250-mM NaCl gradient at 5 ml min^-1^ (Supplementary Figure S5).

### Purification of untagged cyt *c*_6_

The much lower surface charge density of the *Synechocystis* redox carrier proteins necessitated a different purification strategy. Instead of adding precipitation buffer, the periplasmic fraction containing cyt *c*_6_ was clarified a second time at 35,000× ***g*** for 30 min, then passed through a 0.22-µm filter (Sartorius) and concentrated to ~10 ml in a 3-kDa MWCO spin concentrator (ThermoFisher). The concentrated cytochrome solution was then loaded onto a Hiprep 26/60 Sephacryl S-200 HR column (Cytiva) equilibrated in 50 mM CHES at pH 9 with 200 mM NaCl and run at 1.3 ml min^-1^, collecting 5 ml fractions. Any red/pink fractions were pooled and diluted ten-fold in 50 mM Tris pH 9, and then loaded onto a 30-ml Q Sepharose column equilibrated in the same buffer. Anion exchange was then performed to purify cyt *c*_6_ further, using a 30–80 mM NaCl gradient and a flow rate of 5 ml min^-1^ (Supplementary Figure S5).

### Purification of Pc from *Rba. sphaeroides*

With the lowest affinity for ion exchange columns, Pc was the most challenging and time-consuming redox carrier to purify from *Rba. sphaeroides*. The redox carrier protein could only be purified from 1.6-L cultures of evolved *petE* transconjugant strains, by first lysing the cells by French press as described earlier. Clarified lysate was then concentrated to ~2 ml in a 3-kDa MWCO spin concentrator (Sartorius) and oxidised by addition of a few grains of potassium ferricyanide. The 2 ml of oxidised cell lysate was then loaded onto a HiLoad® 16/600 Superdex® 200 pg gel filtration column (Cytiva) equilibrated in salt-free 50 mM Tris-HCl pH 9. As this column was run at 0.5 ml min^-1^, a blue Pc band became visible, along with red and yellow bands. All blue fractions were collected, pooled and diluted ten-fold in salt-free 50 mM Tris-HCl buffer pH 9, and then loaded onto a 30-ml Q Sepharose column for anion exchange over a 25–75 mM NaCl gradient, collecting 5 ml fractions in tubes each containing a small amount of ferricyanide. Once Pc-containing fractions were spectrally identified, pooled and concentrated, a second gel filtration chromatography step was necessary to obtain the desired level of purity, using a Superdex® 75 10/300 GL column (Cytiva) with a running buffer containing 50 mM Tris-HCl pH 8 with 1 M NaCl and a flow rate of 0.3 ml min^-1^. Untagged Pc was required to verify the full activity of a Strep-tagged isoform from *E. coli* which could be produced in higher yields (see below).

### Purification of StrepII-tagged Pc from *E. coli*

To produce enough Pc for kinetic studies, we purified recombinant C-terminally StrepII-tagged Pc produced in *E. coli*. One litre of *E. coli* BL21(DE3) cells transformed with pET28a::*petE*-StrepII was grown at 37°C in a baffled flask with 180-rpm agitation. When the OD_600_ reached 0.6, *petE* expression was induced by adding isopropyl β-D-1-thiogalactopyranoside (IPTG) to 0.5 mM, along with CuSO_4_ to a final concentration of 600 µM. Induced cells were incubated at 37°C overnight with 180-rpm agitation and harvested the next day at 4000× ***g*** for 30 min at 4°C, and then resuspended in 80 ml 20 mM Tris-HCl pH 7.4 with 250 mM DNAse I, and two tablets of EDTA-free protease inhibitor were added. Sodium deoxycholate was then added to a final concentration of 0.1% (w/v), and the cell suspension was mixed by magnetic stirring for 1 h at room temperature before spheroplasts were removed by centrifugation at 8000× ***g*** for 15 min at 4°C. The supernatant was retained, and after addition of several grains of potassium ferricyanide, the periplasmic fraction turned blue and was applied to a 4-ml Strep-Tactin XT 4 Flow column (IBA Lifesciences). The column was washed with 20 mM Tris-HCl pH 7.4, and pure Pc was eluted with a column volume of the same buffer with 50 mM biotin (Supplementary Figure S5). No statistically significant difference in turnover rate was found between untagged and Strep-tagged Pc in steady-state turnover assays with RC complexes (Supplementary Figure S6).

### Determination of periplasmic protein concentrations, to assess the relative production levels for periplasmic cytochromes and blue copper proteins

Single colonies were grown to the 80-ml (see methods above) stage under semi-aerobic conditions, and the entire culture was pelleted at 4200× ***g*** for 30 min at 4°C, removing the supernatant. Each pellet was then resuspended in 500-µl periplasmic extraction buffer (100 mM HEPES pH 8, 500 mM sucrose and 50 mM NaCl) from a 10-ml stock supplemented with 1 EDTA-free protease inhibitor tablet (Merck). The turbidity of each cell suspension was determined by performing a 200-fold dilution and measuring absorbance at 680 nm. Cells were diluted with periplasmic extraction buffer to make a 1-ml suspension for each strain with a consistent OD_680_ value. 200 µl of a 12% (w/v) sodium deoxycholate stock solution in periplasmic extraction buffer was added to each 1-ml suspension and the tubes were incubated in the dark with gentle agitation for 1 h. Spheroplasts were pelleted in 1.5-ml Eppendorf tubes at 16,000× ***g*** for 30 min at 4°C, and 800 µl of each supernatant was transferred to a fresh tube. To remove the deoxycholate from solution, 200 µl 5× deoxycholate precipitation solution (1 M ammonium acetate at pH 5 and 250 mM MgSO_4_) was added to each 800-µl aliquot and mixed thoroughly by inverting the tubes. Most of the precipitated deoxycholate was removed from solution by centrifugation at 16,000× ***g*** for 1 h at 4°C, whilst 10mM stocks of sodium dithionite and potassium ferricyanide were prepared in distilled water. After the centrifugation step, solutions were prepared containing each clarified periplasmic fraction and either 1 mM sodium dithionite or potassium ferricyanide in a 700-µl volume and centrifuged again in Eppendorf tubes at 16,000× ***g*** for 3 h at 4°C.

The concentration of each redox carrier was determined by spectrophotometry in identical cuvettes using a Cary 60 spectrophotometer (Agilent Technologies) baselined to the extracted periplasm of the negative control strain (Δ*cycA* Δ*cycI* pBBRBB). Periplasmic extracts were prepared from two 80-ml cultures of this double knockout strain to provide a baseline for transconjugant strains making cytochromes or Pc. For cytochromes, the extract was reduced by 1 mM sodium dithionite, whilst the second extract was oxidised with 1 mM potassium ferricyanide to provide a baseline for measuring the absorption spectra of Pc. Extinction coefficients could then be used to determine the relative concentrations of each redox carrier protein in solution. As the oxidised extinction coefficient for Pc is significantly smaller than the reduced extinction coefficients for the cytochromes in this work, small amounts of baseline drift could have a disproportionate impact on concentration estimates for Pc strains. To account for this, rather than using the raw value, the OD_597_ for each Pc periplasmic extraction was calculated using the formula (OD_603_–OD_700_)× 1.35, where 1.35 is equal to OD_597_/OD_700_ of pure Pc. Assuming that the proportion of total molecules localised in the periplasm remains the same for each redox carrier protein with equal uptake into chromatophores, this technique provides a cheap and high-throughput tool for assessing the relative production levels for periplasmic cytochromes and blue copper proteins.

### Pyridine hemochromagen assays

To determine the concentration of iso *c*_2_, for which no published extinction coefficients were available, pyridine hemochromagen assays were carried out using the protocol of Barr and Guo [81], using a reduced extinction coefficient of 30.27 mM^-1^cm^-1^ for the pyr_2_-haem *c* co-ordination complex [82]. Absorbance spectra were collected on a Cary 60 spectrophotometer with a 1-cm pathlength, and extinction coefficients were calculated for 551 nm, the closest whole number to the reduced peak of the heme *c* cofactor in iso *c*_2_. This method gave values as follows: ɛ_551 (red)_=25.6 mM^-1^ cm^-1^, ɛ_551 (ox)_=6.53 mM^-1^ cm^-1^ and therefore ɛ_551 (red - ox)_=19.1 mM^-1^ cm^-1^.

### RC-only and RC-LH1 turnover assays

Turnover assays were conducted under steady-state conditions in a similar fashion to those described in Martin et al. [53], using 3×300 µl solutions containing RC or RC-LH1, soluble redox carrier protein and quinone, in a solution containing 0.03% (w/v) β-DDM and, where necessary, 1 mM sodium D-ascorbate to keep cytochromes reduced. Each pre-reduced soluble redox carrier protein was added to a concentration at least 20-fold higher than the RC/RC-LH1 complex in solution. Quinones were added in excess from ethanol stock solutions to a final assay concentration of 50 µM, using a volume of <2 µl to minimise the amount of ethanol in solution.

Following overnight dark adaptation, each 300 µl reaction mixture was placed in a 3-ml, 1cm pathlength quartz cuvette and monitored at a fixed wavelength using a Cary 60 spectrophotometer (Agilent Technologies). The temperature of the solution was maintained at 25°C by a Cary Single Cell Peltier Accessory (Agilent Technologies). Ten seconds into data recording, excitation energy was delivered via a fibre optic cable from an 810-nm M810F2 LED (Thorlabs Ltd., U.K), typically driven at 100% intensity using a DC2200 controller (Thorlabs Ltd., U.K) for 20–50 s. Depending on how rapidly the signal changed, the data were processed by fitting the linear initial rate over 0.025–5 s, starting from the first data point where the absorbance started dropping continuously. Rates were normalised to e^−^/RC/s by dividing the initial rate by the reduced minus oxidised extinction coefficient for the redox carrier protein (Table 3), and then by the RC or RC-LH1 concentration. All cytochrome extinction coefficients are from either *Rba. sphaeroides* or *Synechocystis* sp. PCC 6803, whilst in the absence of a published extinction coefficient for Pc from *Synechocystis* sp. PCC 6803, the ε_597_ from *Phaseolus vulgaris* (common bean) was used instead [86].

**Table 3:** Extinction coefficients used in the present study

Species	Wavelength	Extinction coefficient, ε (mM^-1^)	Source
RC-LH1	875	3000	[83]
RC	802	288	[84]
Cyt *c*_2_	550	30.8 (reduced) 21.5 (reduced–oxidised)	[20]
Iso *c*_2_	551	25.6 (reduced) 19.1 (reduced–oxidised)	The present study
Cyt *c*_6_	552	24.1 (reduced) 19.5 (reduced–oxidised)	[43] [85]
Pc	597	4.5 (reduced) 4.5 (reduced–oxidised)	[86]
Cyt *c*	550	27.6 (reduced) 21.1 (reduced–oxidised)	[87]
DUQ	278	14 (oxidised in EtOH)	[88]
UQ-2	275	13.7 (oxidised in EtOH)	[88]

Cyt *c*, cytochrome *c*. Cyt *c*_2_, cytochrome *c*_2_. Cyt *c*_6_, cytochrome *c*_6_. DUQ, decylubiquinone. Iso *c*_2_, isocytochrome *c*_2_. Pc, plastocyanin. RC, reaction centre. RC-LH1, reaction centre-light harvesting 1. UQ-2, ubiquinone-2.

## Supplementary Material

Online supplementary material

## Data Availability

Any data not provided within the paper as a table, figure or supplementary file is available from the corresponding authors upon request.

## Abbreviations

ALE: adaptive laboratory evolution
BChl: bacteriochlorophyll
Chl: chlorophyll
DUQ: decylubiquinone
ICM: intracytoplasmic membranes
LDAO: lauryldimethylamine oxide
LED: light-emitting diode
MWCO: molecular weight cut-off
PQ: plastoquinone
PSI: photosystem I
PSII: photosystem II
PSIII: photosystem III
Pc: plastocyanin
RC: reaction centre
RC-LH1: reaction centre-light harvesting 1
*Rba*.: *Rhodobacter*
UQ: Ubiquinone
WT: wildtype
cryo-EM: cryogenic electron microscopy
cyt *bc*_1_: cytochrome *bc*_1_
cyt *b*_6_ *f*: cytochrome *b*_6_ *f*
cyt *c*: cytochrome *c*
cyt *c*_2_: cytochrome *c*_2_
cyt *c*_6_: cytochrome *c*_6_
iso *c*_2_: isocytochrome *c*_2_
vdW: van der Waals
