# Supplementary material for: Cyanobacterial redox carriers support photosynthesis in a purple phototrophic bacterium
Source: Biochem J. 2025 Aug 13;482(16):1123–44. doi: 10.1042/BCJ20253114 (PMC12493188; doi:10.1042/BCJ20253114)
Supplement: Online supplementary material [file bcj-482-16-BCJ20253114-s001.docx]

**Supporting Information for** **Cyanobacterial redox carriers support photosynthesis in a purple phototrophic bacterium**

Adam G.M. Bowie^1^, Andrew Hitchcock^1,2*^, Matthew S. Proctor^1^, Elizabeth C. Martin^1^, David J.K. Swainsbury^3^ and C. Neil Hunter^1*^

^1^Plants, Photosynthesis and Soil, School of Biosciences, University of Sheffield, UK.

^2^Molecular Microbiology: Biochemistry to Disease, School of Biosciences, University of Sheffield, UK.

^3^School of Biological Sciences, University of East Anglia, Norwich Research Park, Norwich, UK.

*Correspondence: C. Neil Hunter (c.n.hunter@sheffield.ac.uk); Andrew Hitchcock (a.hitchcock@sheffield.ac.uk)

Author ORCID IDs 0009-0008-6246-4544 (AGMB), 0000-0001-6572-434X (AH), 0000-0002-1484-850X (MSP), 0000-0001-9600-7298 (ECM), 0000-0002-0754-0363 (DJKS), 0000-0003-2533-9783 (CNH).

**Table of Contents:**

| **Supplementary Table 1** | Primers used in this study | **Page 2** |
| --- | --- | --- |
| **Supplementary Table 2** | Predicted electron transfer rates from redox carrier proteins to RC-LH1 and PSI complexes | **Page 3** |
| **Supplementary Figure 1** | Predicted protein-protein docking affinities by the HADDOCK 2.4 server | **Page 4** |
| **Supplementary Figure 2** | Effect of pH on the initial rate of Cyt *c*_2_ oxidation by the WT RC-LH1 core complex | **Page 5** |
| **Supplementary Figure 3** | Raw data showing the effect of increasing NaCl Concentration on initial rates of redox carrier oxidation by RC-LH1 Complexes | **Page 6** |
| **Supplementary Figure 4** | Light saturation of RC and RC-LH1 complexes in steady state turnover assays with cyt *c*_2_ | **Page 7** |
| **Supplementary Figure 5** | Coomassie-stained SDS-PAGE gel of all purified redox carrier proteins | **Page 8** |
| **Supplementary Figure 6** | Steady-state oxidation rates of untagged and StrepII-tagged Pc isoforms by RC-only complexes | **Page 9** |

**Supplementary Table 1.**

| Name | 5’-3’ Sequence | Sites |
| --- | --- | --- |
| *pufBALM* KO UF | CCG**GAATTC**gagagggtcgtgagagagactg | EcoRI |
| *pufBALM* KO UR | GCGC**TCTAGA**agccatgctatcctccggatcg | XbaI |
| *pufBALM* KO DF | GCGC**TCTAGA**aactgaggagcgatcacaatg | XbaI |
| *pufBALM* KO DR | CCCC**AAGCTT**gcagcagacgcgatccaaaag | HindIII |
| *pufBALM* Scr F | gccctggaccgcatcgtagagg |  |
| *pufBALM* Scr R | Cgaaatcacctcggaacgcact |  |
| *pufL* Fwd | atcgAGATCTATGGCACTGCTCAGCTTCG | BglII |
| *pufM* Rev | atcgACTAGTTCAGTTCAGCGGCGCCATG | BcuI |
| *cycI* KO Scr Fwd | CATTTCGTGAATCCGTCCGAGATCG |  |
| *cycI* KO Up Fwd | CCG**GAATTC**CAACGTGAAGGTGATGCGTCAGG | EcoRI |
| *cycI* KO Up Rev | CATTTCAGCCCTCCAATCTCATGGTCTTCTCCCTTTGCG |  |
| *cycI* KO Down Fwd | CTG**AAGCTT**GCCCACGTTCTCG | HindIII |
| *cycI* KO Scr Rev | GCCACAGGATCTTGCCGTCATTG |  |
| *cycA* Fwd | agct**AGATCT**ATGAAGTTCCAAGTCAAGG | BglII |
| *cycA* Rev | tgac**CTCGAG**TCAGGGCCGGACGGCGA | XhoI |
| *cycI* Fwd | agct**AGATCT**ATGAGATTGACCACCATCC | BglII |
| *cycI* Rev | tgac**GTCGAC**TCAGCCCTCCGCCGGCG | SalI |
| *petJ* (Syn) Fwd | agct**AGATCT**ATGTTTAAATTATTCAACCAAGCTAGC | BglII |
| *petJ* (Syn) Rev | tgac**CTCGAG**CTACCAGCCCTTTTCCGC | XhoI |
| *petJ* (Thermo) Fwd | CGC**GGATCC**ATGAAAAAGCGATTCATTAG | BamHI |
| *petJ* (Thermo) Rev | TGAT**CTCGAG**TTAGCCTGCCCAACCCTTG | XhoI |
| *petE* Fwd | agct**AGATCT**ATGTCTAAAAAGTTTTTAACAATCCTCG | BglII |
| *petE* Rev | tgac**CTCGAG**TTACTCAACGACAACTTTGCCTA | XhoI |
| *petE*-StrepII Fwd | aattaA**CCATGG**GCTCCAAGAAGTTTTTGACAATTTTAGCGG | NcoI |
| *petE-*StrepII Rev | agcaat**CTCGAG**CTACTTCTCAAATTGGGGGTGACTCCACGCCGA | XhoI |

**List of Primers used in this study.** Restriction sites used for cloning are underlined in bold.

**Supplementary Table 2.**

*
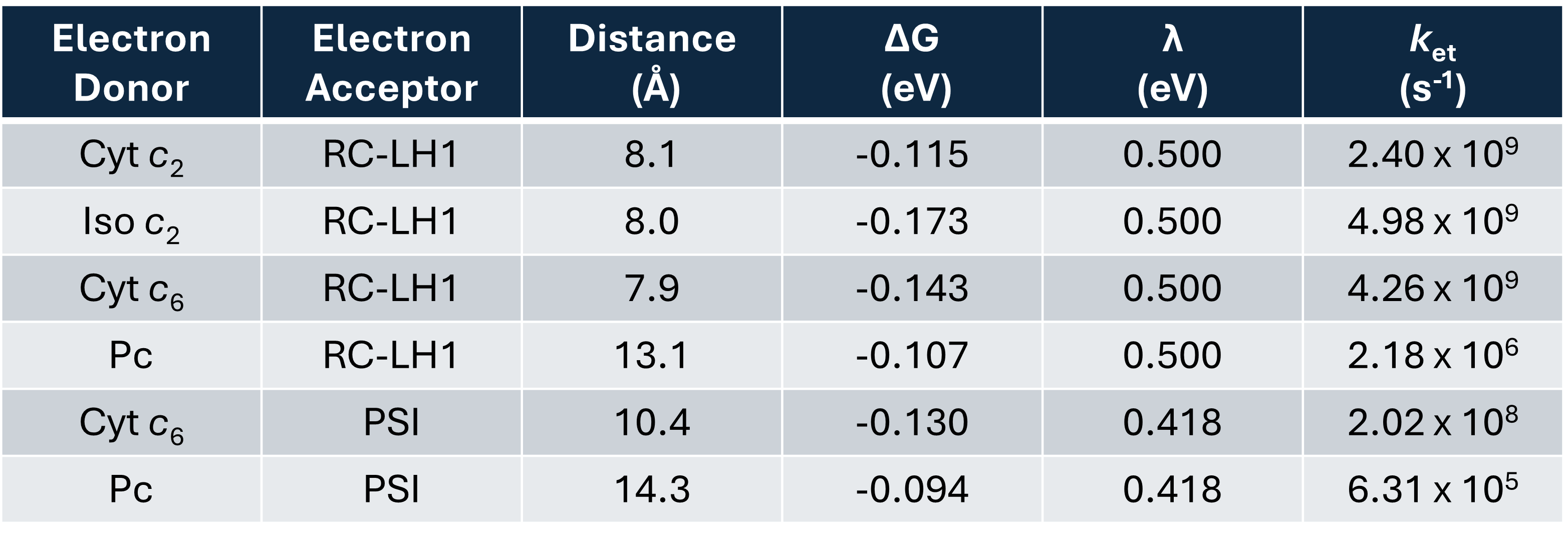
***Predicted electron transfer rates from redox carrier proteins to RC-LH1 and PSI complexes**. Calculations were made using the following equation (Moser et al., 2010), where R is the electron transfer distance in angstroms (Å), ΔG is the free energy change in electron volts (eV) and λ is the reorganisation energy, also in eV.

$${log}_{10}k_{et}^{ex}=13-0.6\left( R-3.6 \right)-3.1(\Delta G+ {\lambda)}^{2}/ \lambda$$

Electron transfer distances represent edge-to-edge cofactor distances and were calculated from AlphaFold3 models (Abramson et al., 2024), whilst ΔG values were computed from midpoint redox potentials for P_865_/P_865_^+^ (Visschers et al., 1999), P_703_/P_703_^+^ (Nakamura et al., 2011), cyt *c*_2_/cyt *c*_2_^+^ (Pettigrew et al., 1976), iso *c*_2_/iso *c*_2_^+^ (Rott et al., 1992), cyt *c*_6_/cyt *c*_6_^+^ and Pc/Pc^+^ (Diaz et al., 1994). Reorganisation energies of +0.5 eV and +0.418 eV were used for electron transfers to RC-LH1 (Lin et al., 1994) and PSI (Caspy et al., 2021), respectively.

**Supplementary Figure 1.**

**
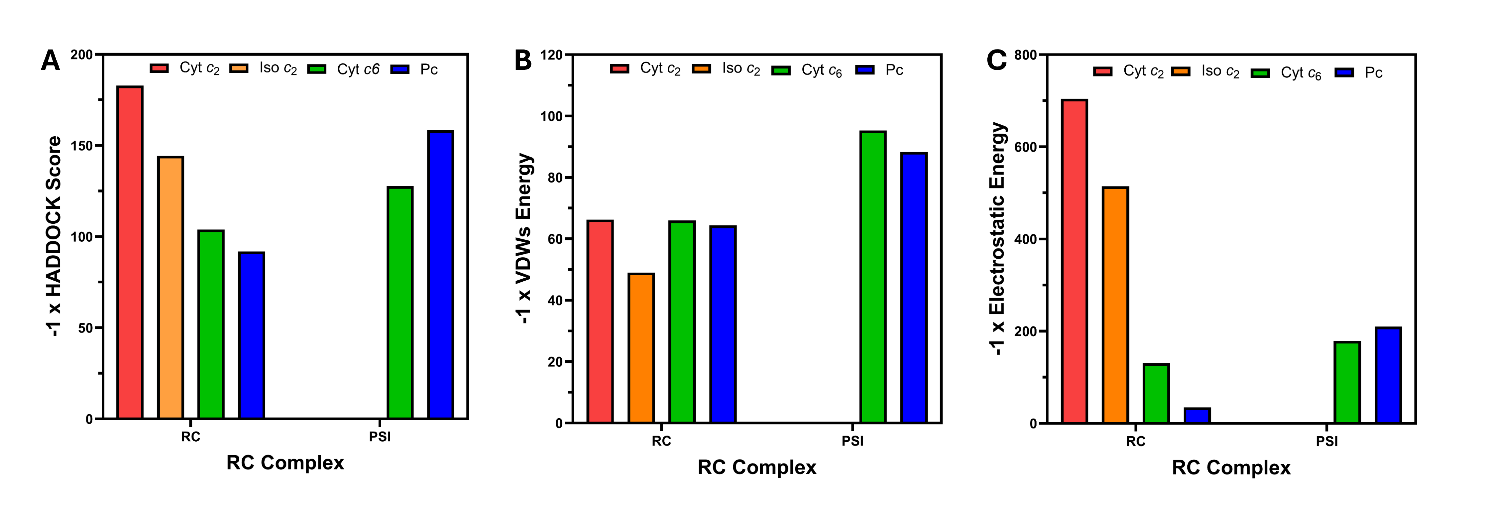
**

**Predicted protein-protein docking affinities by the HADDOCK 2.4 server.** Bar charts of **(A)** Haddock scores, **(B)** van der Waals (VDW) energies and **(C)** electrostatic energies of the native and non-native RC-redox carrier complexes, calculating using the HADDOCK 2.4 server running v2.4-2022.08 (Honorato et al., 2024). For simplicity, all values displayed in the bar charts have been transformed from negative to positive values by multiplying by -1. The output of the HADDOCK 2.4 docking tool is a “HADDOCK score”, which is a weighted sum of the contributing energy components, including VDW forces, electrostatic interactions, desolvation energies and restraint violation energies. This headline score is a model of free energy (ΔG) but is not directly comparable to experimentally determined values.

**Supplementary Figure 2.**


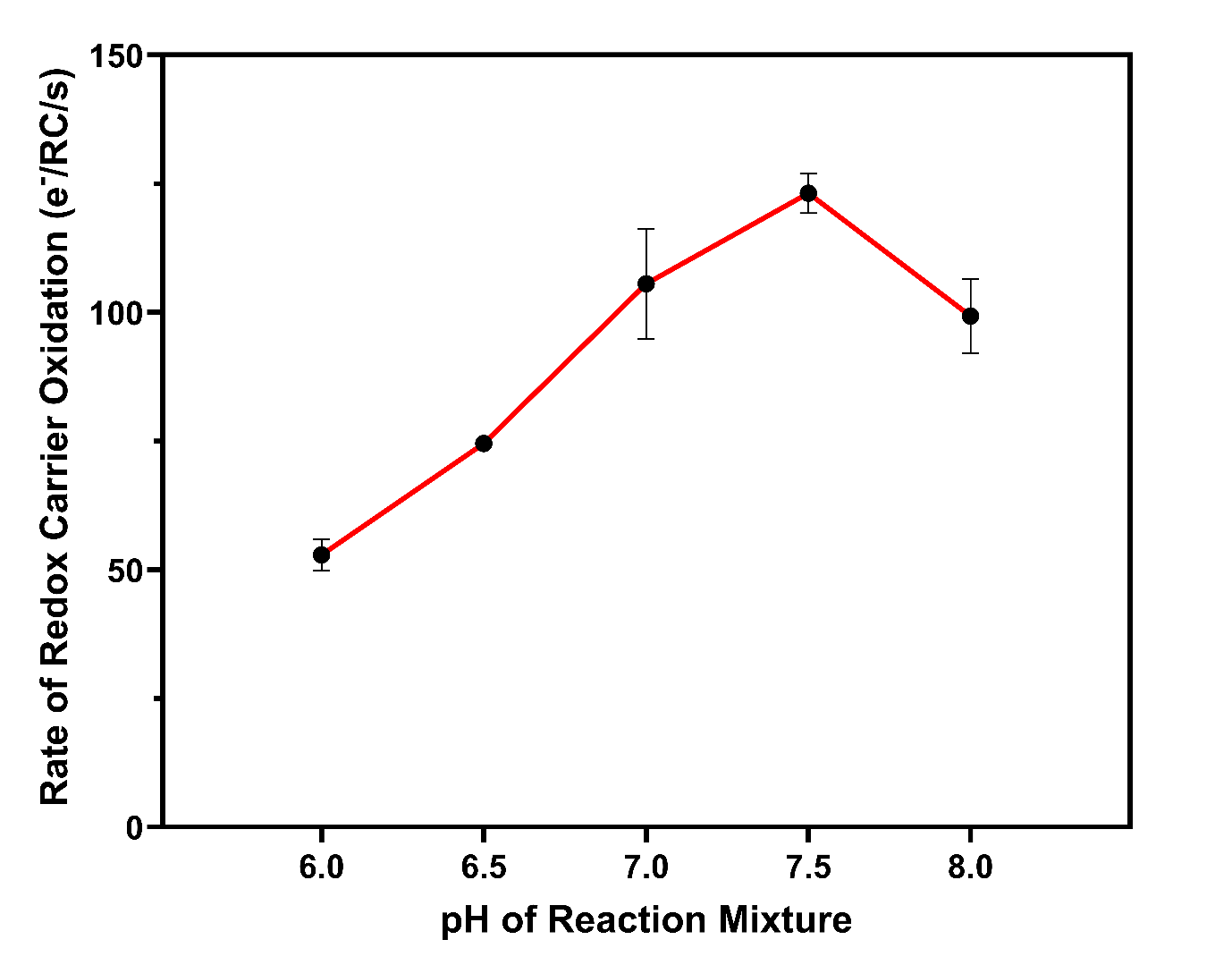
**Effect of pH on the initial rate of cyt *c*_2_ oxidation by the WT RC-LH1 core complex.** To determine the optimum pH value for the RC-LH1 cyt *c*_2_ system, a set of steady-state turnover assays were conducted in triplicate over a range of buffer conditions between pH 6 and 8. Each reaction mixture contained 0.5 µM RC-LH1, 10 µM cyt c2, 500 µM DUQ, 200 mM NaCl, 0.03 % w/v and 100 mM of either Bis-Tris (pH 6 – 6.5), HEPES (pH 7 – 7.5) or Tris-HCl (pH 8) buffers.

**Supplementary Figure 3.**

**
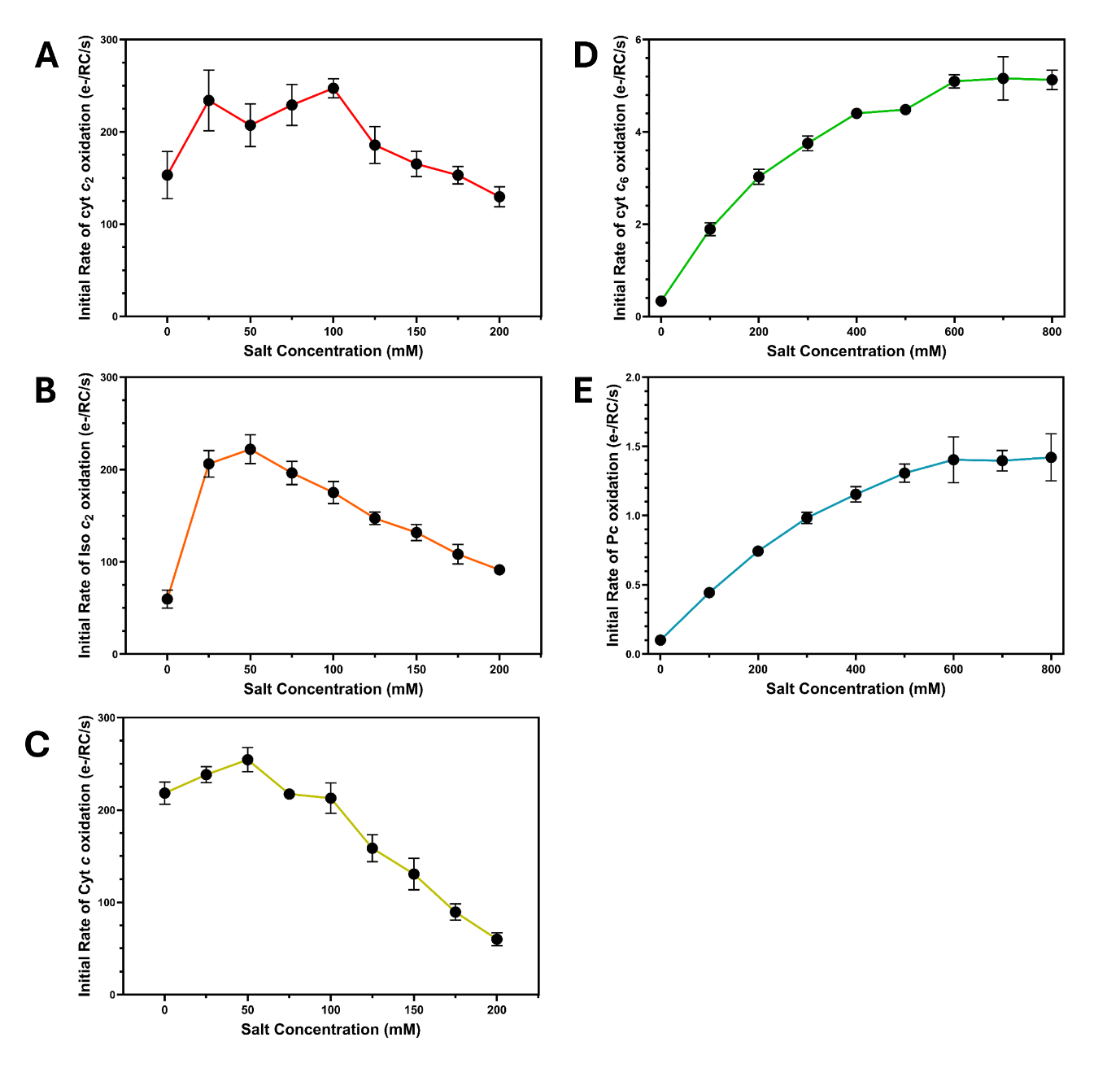
**

**Raw data showing the effect of increasing NaCl Concentration on initial rates of redox carrier oxidation by RC-LH1 Complexes.** Salt dependency of the interaction between RC-LH1 and **(A)** cyt c2 from Rba. sphaeroides, **(B)** iso c2 from Rba. sphaeroides, **(C)** cyt c from Equus caballus, **(D)** cyt c6 from Synechocystis sp. PCC 6803 and **(E)** Pc from Synechocystis sp. PCC 6803. These graphs show the raw turnover rates that are also presented in Figure 6 of the main text, with error bars showing mean and standard deviation.

**Supplementary Figure 4.**


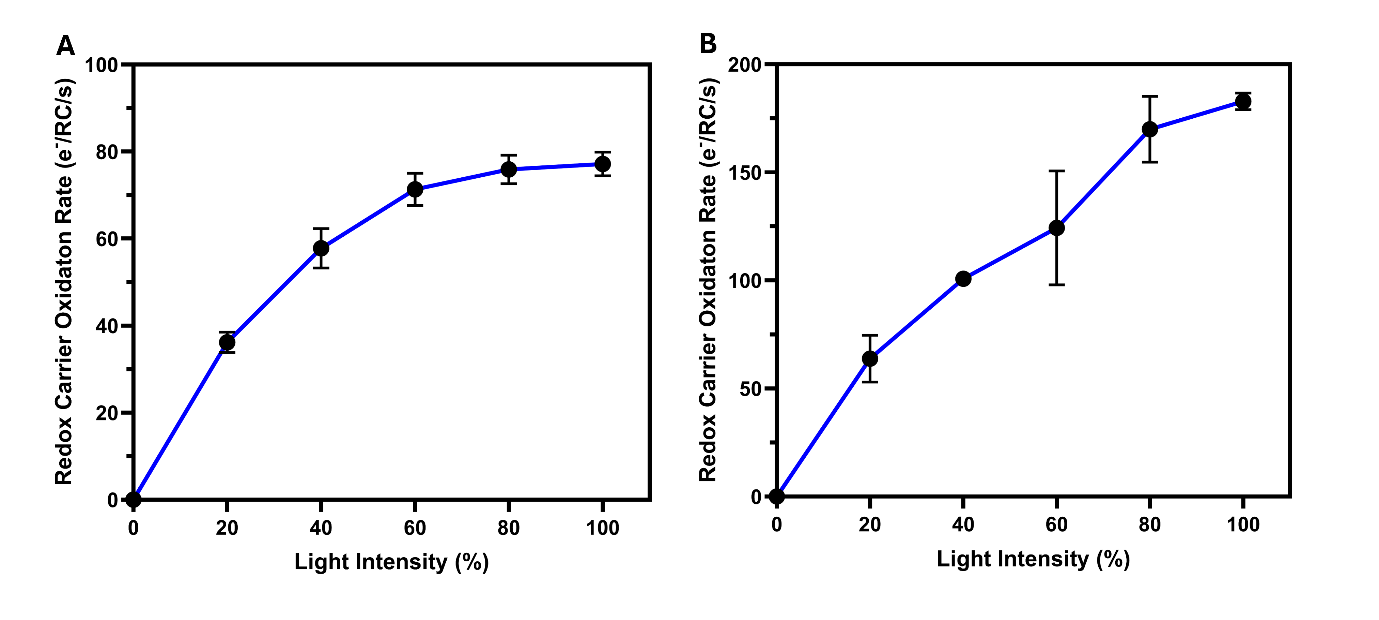


**Light saturation of RC and RC-LH1 complexes by 810 nm LED in steady state turnover assays with cyt *c*_2_.** RC and RC-LH1 complexes were assayed in slightly different reaction mixtures, but both under the same conditions of 50 mM Tris-HCl buffer pH 7.5 with 100 mM NaCl and 0.03 % w/v β-DDM. **(A)** Cyt *c*_2_ oxidation rate in reaction mixtures containing 0.5 µM RC, 10 µM cyt *c*_2_ and 500 µM DUQ. **(B)** Cyt *c*_2_ oxidation rate in reaction mixtures containing 0.5 µM RC-LH1, 1 mM sodium ascorbate, 10 µM cyt *c*_2_ and 50 µM UQ-2. The results clearly show that at 100 % intensity, the red-light pulse is saturating for the RC-only complex, and despite greater experimental noise, saturating or nearly saturating for RC-LH1. Therefore, it can be assumed that any observed oxidation rate differences are not caused the greater light harvesting activity of the physiological complex.

**Supplementary Figure 5.**


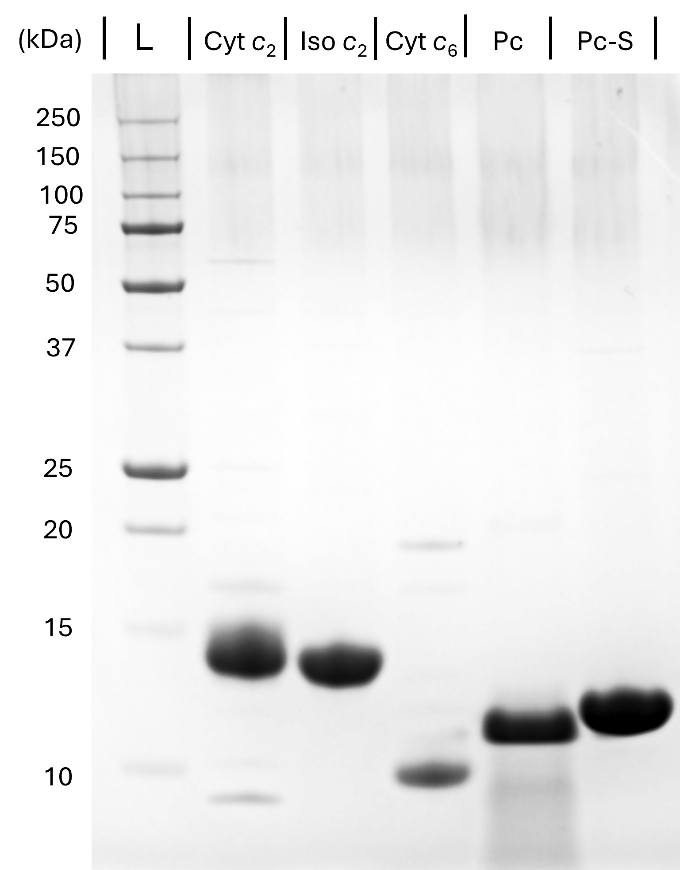


**Coomassie-stained SDS-PAGE gel of all purified redox carrier proteins.** From left to right, cyt *c*_2_ (13.5 kDa) from *Rba. sphaeroides*, iso *c*_2_ (12.9 kDa) from *Rba. sphaeroides*, cyt *c*_6_ (8.7 kDa) from *Rba. sphaeroides*, Pc (10.3 kDa) from *Rba. sphaeroides* and Strep-tagged Pc (11.5 kDa) from *E. coli*.

**Supplementary Figure 6.**


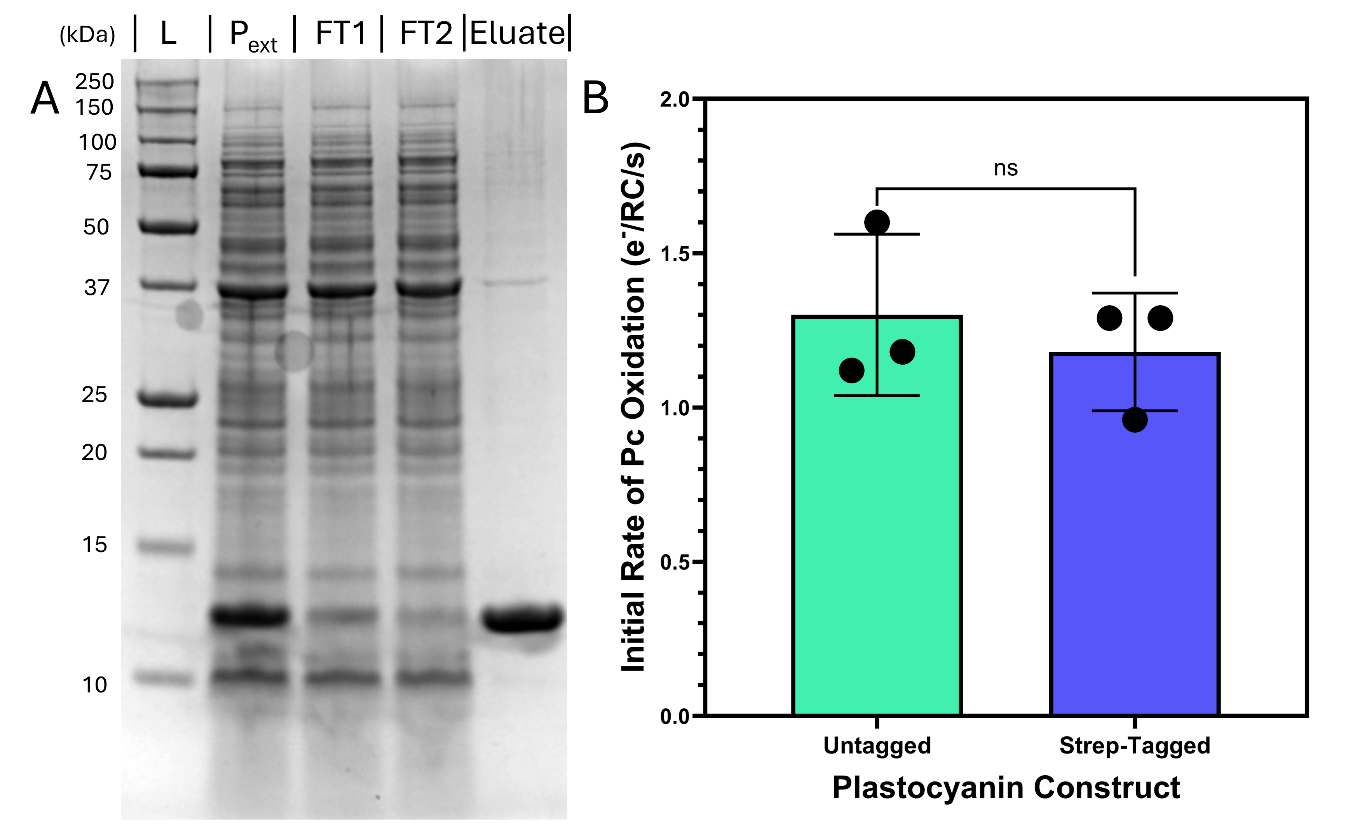


**Steady-state oxidation rates of untagged and StrepII-tagged Pc isoforms by RC-only complexes.** To test if the presence of the StrepII tag made any difference to the oxidation kinetics, steady-state turnover assays were conducted using both tagged and untagged isoforms of the protein in reaction mixtures containing 0.125 µM RC, 10 µM Pc and 50 µM UQ-2 in turnover buffer (50 mM Tris pH 7.5, 100 mM NaCl and 0.03 % (w/v) β-DDM). These assays conclusively showed no difference between the two versions of Pc, with overlapping error bars and a p-value of 0.56 (ns) obtained using an unpaired t test with Welch’s correction applied.
